# Supplementary material for: Community-based complex interventions to sustain independence in older people, stratified by frailty: a protocol for a systematic review and network meta-analysis
Source: BMJ Open. 2021 Feb 15;11(2):e045637. doi: 10.1136/bmjopen-2020-045637 (PMC7887376; doi:10.1136/bmjopen-2020-045637)

Appendix A: MEDLINE search strategy

Database: Ovid MEDLINE(R) and Epub Ahead of Print, In-Process & Other Non-Indexed Citations and Daily <1946 to December 31, 2019>

Search Strategy:

----

1 randomized controlled trial.pt. (497917)

2 controlled clinical trial.pt. (93495)

3 randomized.ab. (465667)

4 placebo.ab. (204054)

5 clinical trials as topic.sh. (189709)

6 randomly.ab. (324606)

7 trial.ti. (210688)

8 or/1-7 (1260622)

9 exp animals/ not humans.sh. (4659772)

10 8 not 9 [Cochrane Highly Sensitive Search Strategy for identifying randomized trials in MEDLINE: sensitivity- and precision-maximizing version (2008 revision] (1159627)

11 Clinical Trial, Phase III/ (16136)

12 ("phase 3" or "phase3" or "phase III" or "P3" or "PIII").ti,ab,kw. (64901)

13 11 or 12 [search filter for phase three trials to suppplement Cochrane HSSS, Cooper 2019] (70728)

14 10 or 13 [final RCT filter] (1199211)

15 (frail* or prefrailty).tw. (19992)

16 exp aged/ (3036862)

17 geriatrics/ (29616)

18 (elder* or older or old people* or old person* or old wom#n*1 or old m#n*1 or old male*1 or old female*1 or old adult*1 or old age* or aging or ageing or geriatric* or senior citizen* or seniors or pensioner* or veteran* or sexagenarian* or septuagenarian* or octogenarian* or nonagenarian* or centenarian*).tw,kf. (1233323)

19 (over adj2 ("60" or "61" or "62" or "63" or "64" or "65" or "66" or "67" or "68" or "69" or "70" or "71" or "72" or "73" or "74" or "75" or "76" or "77" or "78" or "79" or "80" or "81" or "82" or "83" or "84" or "85" or "86" or "87" or "88" or "89" or "90" or "91" or "92" or "93" or "94" or "95" or "96" or "97" or "98" or "99" or "100") adj years).tw. (19080)

20 or/15-19 [older or frail people] (3792854)

21 independent living/ (5105)

22 community health services/ (31116)

23 community health nursing/ (19479)

24 Community support services.tw. (163)

25 exp managed care programs/ (39897)

26 (health maintenance organi?ation* or HMO*).tw. (13072)

27 (Social adj3 services).tw. (9360)

28 Voluntary services.tw. (93)

29 *home nursing/ (5264)

30 House Calls/ (3475)

31 house call*.tw. (615)

32 (home adj5 visit*).tw. (11016)

33 ((general practice or primary care or nurse* or group or ambulatory clinic or geriatric clinic) adj3 visit*).tw. (8476)

34 *geriatric assessment/ (12510)

35 (pharmac* adj2 visit).tw. (182)

36 ((home or house) adj2 appointment*).tw. (42)

37 Home Care Services/ (32888)

38 Home care service*.tw. (1725)

39 *health services for the aged/ (13598)

40 home health nursing/ (319)

41 district nursing.tw. (649)

42 health visit*.ti. or health visit*.ab. /freq=2 (2224)

43 community matron*.ti. or community matron*.ab. /freq=2 (83)

44 (home adj3 (intervention* or support* or assessment*)).tw. (7580)

45 preventive health services/ (13212)

46 ((preventive* or preventative*) adj5 medicine).tw. (6792)

47 preventative medicine/ (11603)

48 ((preventive* or preventative*) adj3 (program* or intervent* or support* or care or service* or approach* or case management or measure* or OT or occupational therapy or assess*)).tw. (57977)

49 or/21-48 [specific interventions] (261556)

50 geriatric nursing/ (13511)

51 geriatric nurs*.tw,kf. (1086)

52 or/50-51 [geriatric nursing] (13877)

53 community.ti,ab,kf. (464179)

54 community health services/ or community health nursing/ or community mental health services/ or community pharmacy services/ (71636)

55 "domiciliary care"/ (32888)

56 aftercare/ (8799)

57 primary health care/ (75131)

58 (domiciliary or (social support and home*) or ((homecare or medical) adj2 home) or (home and package*) or (outreach and home) or (alternative setting and home) or home visit* or home manag* or homecare or home care or home therap* or (model* adj1 home*) or home program* or home monitor*).tw. (53213)

59 ((live or living or lived or dwell*) adj5 ("at home" or "own home" or "in home" or alone or independent*)).tw. (15304)

60 (home-based or homebased or homebound).tw. (10615)

61 (Home care or primary care or primary health care or primary healthcare).tw. (146114)

62 or/53-61 [interventions in a community or home setting] (709468)

63 52 and 62 [geriatric nursing and interventions in a community or home setting] (1982)

64 49 or 63 [all interventions] (262247)

65 (coronary heart disease or CHD or chronic obstructive pulmonary disease or COPD or kidney failure or CKD or Heart failure or diabetes or asthma or cancer or schizophrenia or severe mental illness*).ti. (1420118)

66 64 not 65 [all intervenions excluding specific diseases in title] (247185)

67 14 and 20 and 66 [RCTs and older people and interventions] (6170)

Figure S1: PRISMA flow chart template for NIHR128862

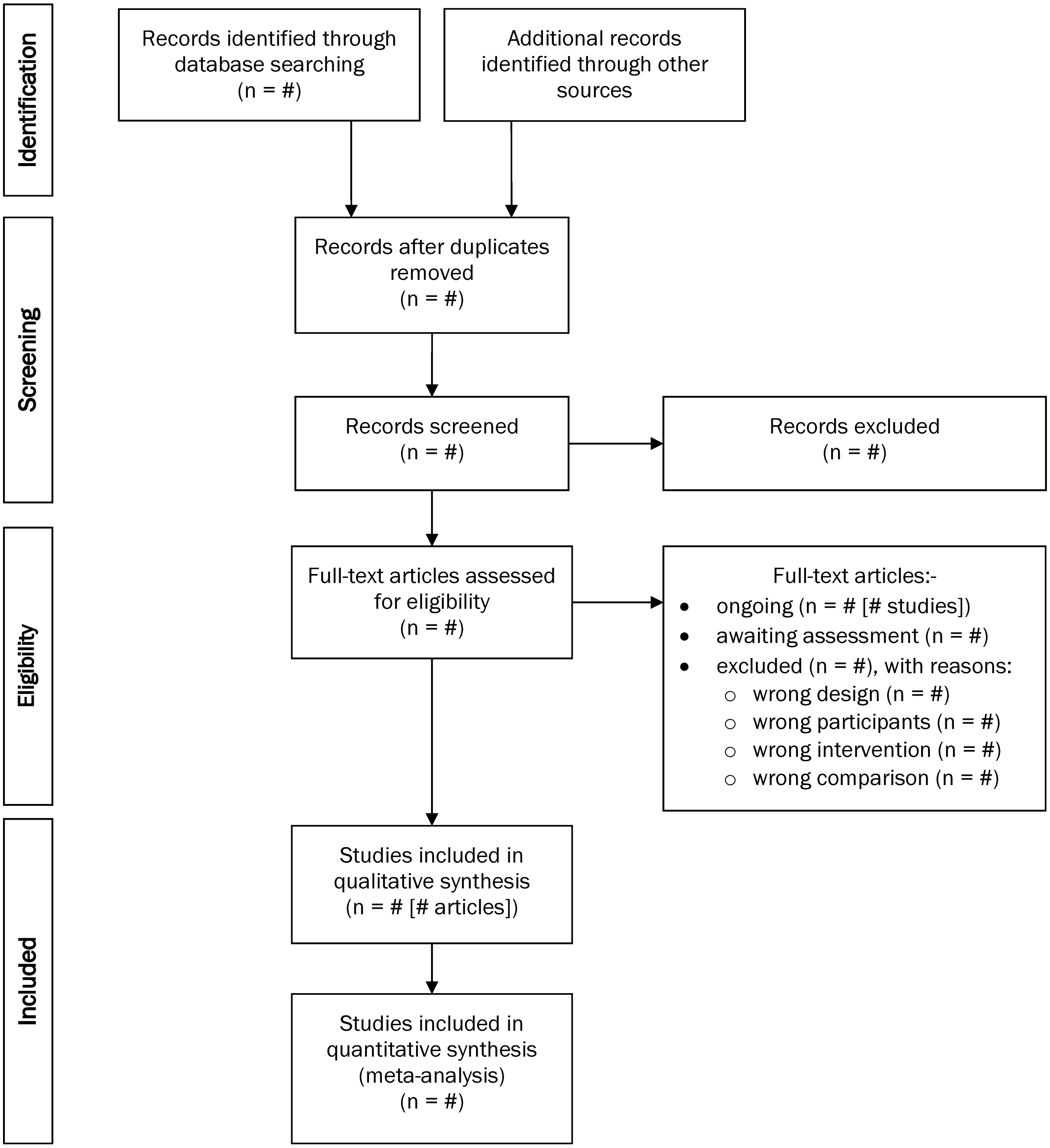

Supplement: Supplementary data [file bmjopen-2020-045637supp001.pdf]
