# Supplementary material for: Community-based complex interventions to sustain independence in older people, stratified by frailty: a protocol for a systematic review and network meta-analysis
Source: BMJ Open. 2021 Feb 15;11(2):e045637. doi: 10.1136/bmjopen-2020-045637 (PMC7887376; doi:10.1136/bmjopen-2020-045637)
Supplement: Author's manuscript [file bmjopen-2020-045637.draft_revisions.pdf]

BMJ Open is committed to open peer review. As part of this commitment we make the peer review history of every article we publish publicly available.

When an article is published we post the peer reviewers' comments and the authors' responses online. We also post the versions of the paper that were used during peer review. These are the versions that the peer review comments apply to.

The versions of the paper that follow are the versions that were submitted during the peer review process. They are not the versions of record or the final published versions. They should not be cited or distributed as the published version of this manuscript.

BMJ Open is an open access journal and the full, final, typeset and author-corrected version of record of the manuscript is available on our site with no access controls, subscription charges or pay-per-view fees (http://bmjopen.bmj.com).

If you have any questions on BMJ Open's open peer review process please email info.bmjopen@bmj.com

BMJ Open

Community-based complex interventions to sustain independence in older people, stratified by frailty: a protocol for a systematic review and network meta-analysis

Journal:	*BMJ Open*
Manuscript ID	bmjopen-2020-045637
Article Type:	Protocol
Date Submitted by the Author:	08-Oct-2020
Complete List of Authors:	Crocker, Thomas; Bradford Teaching Hospitals NHS Foundation Trust, Academic Unit for Ageing and Stroke Research<br>Clegg, Andrew; University of Leeds, Academic Unit for Ageing and Stroke Research, Bradford Institute for Health Research, Bradford Teaching Hospitals NHS Foundation Trust<br>Riley, Richard; Keele University, Centre for Prognosis Research, School of Medicine<br>Lam, Natalie; Bradford Teaching Hospitals NHS Foundation Trust, Bradford Institute for Health Research, Academic Unit for Ageing and Stroke Research<br>Bajpai, Ram; Keele University, School of Medicine; University of Leeds Leeds Institute of Health Sciences<br>Jordão, Magda; Bradford Teaching Hospitals NHS Foundation Trust, Bradford Institute for Health Research, Academic Unit for Ageing and Stroke Research<br>Patetsini, Eleftheria; Bradford Teaching Hospitals NHS Foundation Trust, Bradford Institute for Health Research, Academic Unit for Ageing and Stroke Research<br>Ramiz, Ridha; Bradford Teaching Hospitals NHS Foundation Trust, Bradford Institute for Health Research, Academic Unit for Ageing and Stroke Research<br>Ensor, Joie; Keele University, Centre for Prognosis Research, School of Medicine<br>Forster, Anne; University of Leeds, Academic Unit for Ageing and Stroke Research, Bradford Institute for Health Research, Bradford Teaching Hospitals NHS Foundation Trust<br>Gladman, John; University of Nottingham, Nottingham University Hospitals NHS Trust, NIHR Applied Research Collaboration (ARC) East Midlands, NIHR Nottingham Biomedical Research Centre
Keywords:	GERIATRIC MEDICINE, Clinical trials < THERAPEUTICS, EPIDEMIOLOGY

SCHOLARONE™
Manuscripts

I, the Submitting Author has the right to grant and does grant on behalf of all authors of the Work (as defined in the below author licence), an exclusive licence and/or a non-exclusive licence for contributions from authors who are: i) UK Crown employees; ii) where BMJ has agreed a CC-BY licence shall apply, and/or iii) in accordance with the terms applicable for US Federal Government officers or employees acting as part of their official duties; on a worldwide, perpetual, irrevocable, royalty-free basis to BMJ Publishing Group Ltd ("BMJ") its licensees and where the relevant Journal is co-owned by BMJ to the co-owners of the Journal, to publish the Work in this journal and any other BMJ products and to exploit all rights, as set out in our licence.

The Submitting Author accepts and understands that any supply made under these terms is made by BMJ to the Submitting Author unless you are acting as an employee on behalf of your employer or a postgraduate student of an affiliated institution which is paying any applicable article publishing charge ("APC") for Open Access articles. Where the Submitting Author wishes to make the Work available on an Open Access basis (and intends to pay the relevant APC), the terms of reuse of such Open Access shall be governed by a Creative Commons licence – details of these licences and which Creative Commons licence will apply to this Work are set out in our licence referred to above.

Other than as permitted in any relevant BMJ Author's Self Archiving Policies, I confirm this Work has not been accepted for publication elsewhere, is not being considered for publication elsewhere and does not duplicate material already published. I confirm all authors consent to publication of this Work and authorise the granting of this licence.

For peer review only

Community-based complex interventions to sustain independence in older people, stratified by frailty: a protocol for a systematic review and network meta-analysis

Thomas F. Crocker, Andrew Clegg, Richard D. Riley, Natalie Lam, Ram Bajpai, Magda Jordão, Eleftheria Patetsini, Ridha Ramiz, Joie Ensor, Anne Forster, John R.F. Gladman

Thomas F. Crocker
1. Academic Unit for Ageing and Stroke Research, Bradford Institute for Health Research, Bradford Teaching Hospitals NHS Foundation Trust, Bradford, UK
Email: tom.crocker@bthft.nhs.uk ; ORCID: 0000-0001-7450-3143

Andrew Clegg
1. Academic Unit for Ageing and Stroke Research, University of Leeds, Bradford Institute for Health Research, Bradford Teaching Hospitals NHS Foundation Trust, Bradford, UK
Email: a.p.clegg@leeds.ac.uk ; ORCID: 0000-0001-5972-1097

Richard D. Riley
1. Centre for Prognosis Research, School of Medicine, Keele University, Keele, UK
Email: r.riley@keele.ac.uk ; ORCID: 0000-0001-8699-0735

Natalie Lam
1. Academic Unit for Ageing and Stroke Research, Bradford Institute for Health Research, Bradford Teaching Hospitals NHS Foundation Trust, Bradford, UK
Email: natalie.lam@bthft.nhs.uk ; ORCID: 0000-0001-8591-444X

Ram Bajpai
1. School of Medicine, Keele University, Keele, UK
2. Leeds Institute of Health Sciences, University of Leeds, Leeds, UK
Email: r.bajpai@keele.ac.uk ; ORCID: 0000-0002-1227-2703

Magda Jordão
1. Academic Unit for Ageing and Stroke Research, Bradford Institute for Health Research, Bradford Teaching Hospitals NHS Foundation Trust, Bradford, UK
Email: magda.jordao@bthft.nhs.uk ; ORCID: 0000-0003-2108-2677

Eleftheria Patetsini
1. Academic Unit for Ageing and Stroke Research, Bradford Institute for Health Research, Bradford Teaching Hospitals NHS Foundation Trust, Bradford, UK
Email: eleftheria.patetsini@bthft.nhs.uk ; ORCID: 0000-0001-8393-982X

Ridha Ramiz
1. Academic Unit for Ageing and Stroke Research, Bradford Institute for Health Research, Bradford Teaching Hospitals NHS Foundation Trust, Bradford, UK
Email: ridha.ramiz@bthft.nhs.uk ; ORCID: 0000-0002-3569-8731

Joie Ensor
1. Centre for Prognosis Research, School of Medicine, Keele University, Keele, UK
Email: j.ensor@keele.ac.uk ; ORCID: 0000-0001-7481-0282

Anne Forster
1. Academic Unit for Ageing and Stroke Research, University of Leeds, Bradford Institute for Health Research, Bradford Teaching Hospitals NHS Foundation Trust, Bradford, UK
Email: a.forster@leeds.ac.uk ; ORCID: 0000-0001-7466-4414

John R.F. Gladman

For peer review only - http://bmjopen.bmj.com/site/about/guidelines.xhtml

1. University of Nottingham, Nottingham, UK
2. Nottingham University Hospitals NHS Trust, Nottingham, UK
3. NIHR Applied Research Collaboration (ARC) East Midlands, UK
4. NIHR Nottingham Biomedical Research Centre, UK
   Email: john.gladman@nottingham.ac.uk ; ORCID: 0000-0002-8506-7786

Corresponding author:

Thomas F. Crocker
Academic Unit for Ageing and Stroke Research, Bradford Institute for Health Research, Bradford Teaching Hospitals NHS Foundation Trust, Bradford, UK
Email: tom.crocker@bthft.nhs.uk ; ORCID: 0000-0001-7450-3143

Word counts:

Abstract: 297

Manuscript: 3,894

For peer review only

For peer review only - http://bmjopen.bmj.com/site/about/guidelines.xhtml

Abstract

Introduction

Maintaining independence is a primary goal of community health and care services for older people but there is currently insufficient guidance about which services to implement. Therefore, we aim to synthesise evidence on the effectiveness of community-based complex interventions to sustain independence for older people, including the effect of frailty, and group interventions to identify the best configurations.

Methods and analysis

Systematic review and network meta-analysis (NMA). We will include randomised controlled trials (RCTs) and cluster RCTs of community-based complex interventions for older people living at home (mean age ≥ 65 years), compared with usual care or another complex intervention. We will search MEDLINE, Embase, CENTRAL, CINAHL, PsycINFO and clinical trial registries from inception, without language restrictions, and scan reference lists of included papers. The main outcomes are living at home, activities of daily living (basic/instrumental), home-care services usage, hospitalisation, care home admission, costs and cost-effectiveness. Additional outcomes are health status, depression, loneliness, falls and mortality. Interventions will be coded, summarised and grouped. An NMA using a multivariate random-effects model for each outcome separately will determine the relative effects of different complex interventions. For each outcome, we will produce summary effect estimates for each pair of treatments in the network, with 95% confidence interval, ranking plots and measures, and the borrowing of strength statistic. Inconsistency will be examined using a 'design-by-treatment interaction' model. We will assess risk of bias (Cochrane tool, version 2) and certainty of evidence using the Grading of Recommendations Assessment, Development and Evaluation for NMA approach.

Ethics and dissemination

This systematic review will use aggregated, anonymised, publicly available data. Findings will be reported according to Preferred Reporting Items for Systematic Reviews and Meta-analyses guidance. They will be disseminated widely to policymakers, commissioners and providers, presented at international conferences and published in scientific journals.

PROSPERO registration: CRD42019162195

Keywords: Community-based complex intervention, older people, frailty, Network meta-analysis, Systematic review, Randomised controlled trial, ageing in place, resilience

For peer review only - http://bmjopen.bmj.com/site/about/guidelines.xhtml

Strengths and limitations of this study

- This will be the first systematic review with network meta-analysis comparing the effectiveness of community-based complex interventions to sustain independence for older people, including the effect of frailty and pre-frailty.
- A careful process to group interventions, including summarising each intervention with the Template for Intervention Description and Replication (TIDieR), will produce an analysis that is transparent and relevant to policymakers, commissioners and providers.
- In addition to the direct treatment effects, indirect treatment effects will be analysed using a random-effects network meta-analysis allowing us to compare different service models with each other.
- Summary of Findings tables developed using the GRADE approach for NMA will provide an accessible assessment of the certainty and size of treatment effects.
- The review is likely to be limited by lack of detail about the experimental conditions and wider care system in some trials, and the lack of consistent outcome measures.

For peer review only - http://bmjopen.bmj.com/site/about/guidelines.xhtml

Introduction

Global population projections indicate that older people are the fastest growing demographic, with the percentage of people aged 65 and over expected to almost double by 2050[1], and similar projections for developed countries such as the UK.[2] Given this success of increasing lifespan, current policy and initiatives such as the World Health Organization's Decade of Healthy Ageing emphasise increasing the number of years lived *in good health.*[3 4] This focus on sustaining health is crucial for enabling people to realise their strong preference for living with independence within a community[5]. Additionally, older people are core users of health and care services, so the ageing population demographic has profound implications for service planning and delivery. However, there is currently insufficient guidance for policymakers, commissioners and providers about which community services should be implemented.

Frailty is an especially problematic feature of population ageing, with increased risk of losing independence, hospitalisation, care home admission and mortality.[6] In the UK, around 10% of people aged 65 and over have frailty, rising to around 50% of people aged over 85.[7] UK NHS expenditure increases considerably with advancing age, with a threefold increase for people aged over 70.[8] UK social care expenditure for older people is expected to rise to £12.7 billion by 2022.[9] Extra annual cost to the healthcare system per person was £561.05 for mild, £1,208.60 for moderate and £2,108.20 for severe frailty with reference to 2013/14 UK costs.[10] This estimates a total additional cost of £5.8 billion per year across the UK.[10] These findings are mirrored in other developed countries.

There is a critical evidence gap regarding which community-based interventions are clinically and cost-effective, and therefore appropriate, for older people, including those living with frailty and pre-frailty. This evidence gap means that there is considerable uncertainty regarding how interventions should best be configured and commissioned. Previous systematic reviews and meta-analyses have reported evidence for clinical and cost-effectiveness of community-based complex interventions for reducing hospital admission, nursing home admission, falls and functional decline in older people.[11-13] However, previous reviews have not used NMA to summarise whether different types of interventions have differential effects on outcomes, limiting usefulness for policymakers, health and social care commissioners and providers. A landmark 2008 systematic review and meta-analysis summarised evidence from 89 trials including 97,984 people.[11] The review reported that, in general, complex interventions provided in the community are effective for older people but lacked detail about what types of complex care improve outcomes, and does not include studies published over the last decade, which are potentially influential. This review only considered frailty in relation to one intervention (Comprehensive Geriatric Assessment) and used a disability-based,

For peer review only - http://bmjopen.bmj.com/site/about/guidelines.xhtml

non-validated definition of frailty to categorise trials. Standard meta-analysis techniques were used to synthesise the evidence.

Recognising that research evidence, understanding of frailty, and meta-analysis methods have advanced considerably in the last decade the review requires a contemporary update to identify how interventions might best be configured to improve outcomes and inform commissioning and delivery of evidence-based services.

Specific review questions are:

(i) Do community-based complex interventions to sustain independence in older people increase living at home, independence and health-related quality of life?
(ii) Do community-based complex interventions to sustain independence in older people reduce home care requirement, depression, loneliness, falls, hospitalisation, care home admission, costs and mortality?
(iii) How should interventions be grouped for network meta-analysis (NMA)?
(iv) What is the optimal configuration of community-based complex interventions to sustain independence in older people?
(v) Do intervention effects differ by frailty level (not frail; pre-frailty; frailty)?

Objectives

The overall aim of this systematic review is to synthesise evidence on the effectiveness of community-based complex interventions to sustain independence in older people, including the effect of frailty and pre-frailty, and group interventions to identify the best configurations. For this systematic review we define sustaining independence to mean maintaining or improving independence in activities of daily living (washing, dressing, grooming, toileting, walking, preparing meals, doing housework, managing finances, assisting others, etc), but not only one of these specific activities (e.g., walking only). The specific objectives are as follows:

(i) To identify randomised controlled trials (RCTs) and cluster randomised controlled trials (cRCTs) of community-based complex interventions to sustain independence in older people.
(ii) To synthesise evidence of their effectiveness for key outcomes in a meta-analysis of study-level data.
(iii) To identify key intervention components and study-level frailty to inform groupings for NMA and meta-regression.
(iv) To compare effectiveness of different intervention configurations using NMA.
(v) To investigate the impact of frailty and pre-frailty using meta-regression.

For peer review only - http://bmjopen.bmj.com/site/about/guidelines.xhtml

Methods and analysis

This protocol is registered on the International Prospective Register of Systematic Reviews (PROSPERO) (CRD42019162195). This protocol is reported in accordance with the reporting guidance provided in the Preferred Reporting Items for Systematic Reviews and Meta-Analyses Protocols (PRISMA-P) statement (Supplementary materials: Additional file 1) and PRISMA-NMA reporting guidelines.[14 15]

Eligibility criteria

We will select studies according to their design and the PICO criteria: participants, intervention, comparator and outcome(s) of interest.

Study design: randomised controlled trials (RCTs) and cluster randomised controlled trials (cRCTs). Where only one unit of randomisation (an individual or cluster) is allocated to an arm of a trial, we will exclude the trial as the treatment effect is completely confounded with the unit. We accept minimisation as a method of sequence generation, in keeping with the Cochrane risk of bias guidance. Crossover trials are also eligible, however, we will only use outcome data from the pre-crossover period.

Participants (population): older people living at home (mean age of participants 65 years or older). We will exclude trials of residents of care/nursing homes as these are the subject of other large-scale reviews.[16 17] If not all participants are living at home, we will only include the trial if data can be extracted specifically for these participants.

Intervention: Aligned with our focus on community-based complex interventions, trials will be considered eligible if:

- the intervention is both initiated and mainly provided in the community;
- the intervention includes two or more interacting components (intervention practices, structural elements, and contextual factors);
- the intervention is targeted at the individual person, with provision of appropriate specialist care; and
- a focus of the intervention is sustaining (maintaining or improving) the person's independence.

A broad range of interventions will potentially be eligible, which may differ in terms of how the service is organised and also in what is actually done to or for the older person.

Interventions that would not be considered eligible for inclusion are as follows.

For peer review only - http://bmjopen.bmj.com/site/about/guidelines.xhtml

- The intervention is either not initiated, or not mainly provided, in the community, or neither. For example, interventions delivered in outpatient, day hospital, inpatient, and intermediate (post-acute) care settings.
- The intervention includes only one component (intervention practices, structural elements and contextual factors) such as a drug, treadmill training, yoga, provision of information, cataract surgery, hearing aid, medication review, nutritional supplements.
- The intervention is not targeted at the individual person, with provision of appropriate specialist care. For example, general staff education (not training in a patient-level intervention), practice-level reorganisation, operational, managerial or IT interventions, public health messages.
- A focus of the intervention is not sustaining (maintaining or improving) independence in activities of daily living. For example, interventions that primarily address cognitive deficits, mood disorders, or both will be excluded, unless they also aimed to improve overall independence.
- Condition-specific interventions, for example case management for older people with diabetes, COPD or depression.
- Interventions in which the primary focus is falls prevention as this evidence is already well synthesised, including in a recent NMA.[18] Nonetheless, falls will be a key additional outcome in this review.

Comparator: usual care, “placebo” or attention control, or a different complex intervention meeting our criteria.

Outcome(s): Studies will be included where outcome data were recorded at a minimum 24-week timepoint. For all outcomes of interest, data will be extracted and categorised for three timepoints: around 6 months, around 12 months, and around 24 months.

Main outcomes

– The main outcomes are: living at home (defined either as a reported trial outcome, or the inverse of care home admission and mortality if reported separately); activities of daily living (basic/instrumental); home-care services (non-healthcare professional) usage; hospitalisation; care home admission; costs; and cost-effectiveness.

Additional outcomes

– The additional outcomes are: health status/health-related quality of life, depression, loneliness, falls, mortality.

For peer review only - http://bmjopen.bmj.com/site/about/guidelines.xhtml

This update to the landmark 2008 systematic review and meta-analysis by Beswick and colleagues[11] refines the criteria used by that review, which will lead to the exclusion of some of their included studies; we recount these differences here. We will exclude falls prevention studies as a recent network meta-analysis has been conducted in that area.[18] Our criteria exclude interventions that are initiated in hospital and those conducted in outpatient settings, to ensure the interventions are firmly placed in the community. We will also exclude interventions in residential care settings, as these are already the subject of large-scale reviews, and the different settings provide different opportunities and challenges for intervening. Finally, we will exclude studies without an intervention targeted at the older person, for example financial incentives for general practitioners, for consistency within our network meta-analysis.

Search strategy

Search strategies have been developed and tested through an iterative process by an experienced medical information specialist in consultation with the review team. We will search following databases from inception: CENTRAL, MEDLINE, EMBASE, CINAHL, PsycINFO databases. We will also search trial registers (ClinicalTrials.gov and the International Clinical Trials Registry) and scan reference lists of included papers. Publication status/language restrictions will not be used, and translation will be arranged as necessary throughout the process. A draft search strategy for MEDLINE is provided in the *supplementary appendix A*. A Preferred Reporting Items for Systematic Reviews and Meta-Analyses (PRISMA) flow chart will be presented showing the process of study selection (Supplementary figure S1).[14]

Study selection

Following deduplication, search results will be imported into the Rayyan web application (https://rayyan.qcri.org/). Two researchers will independently assess the title and abstract of each record. We will obtain full text articles for all potentially eligible trials. Study selection will be conducted by two researchers with guidance from the project management group (PMG), and disagreements will be resolved by consensus discussion involving the PMG. We will contact study authors if further information is required.

Data collection process

Two researchers will independently extract data using a piloted data extraction form in a purpose-built Microsoft Access database. Characteristics of included and excluded studies tables will be produced in Review Manager (RevMan) [Computer program] Version 5.4. Summary of findings tables will be produced in GRADEPro.[19]

For peer review only - http://bmjopen.bmj.com/site/about/guidelines.xhtml

Intervention grouping

We will group interventions for NMA in a three-stage process.

1. We will use the Template for Intervention Description and Replication (TIDieR) framework to summarise reported interventions (including comparators).[20] The TIDieR framework includes 12 key items including the why, what, who provided, how, where, when and how much of the intervention including the broader healthcare context.
2. We will complete a content analysis of the summarised interventions using the TIDieR framework in nVivo 12 to inform provisional groupings.[21]
3. We will develop provisional intervention groupings based upon the service organisation or structure (e.g., team structure), key patient care processes (e.g., assessment, follow-up), and specific patient care interventions (e.g., exercise, ADL practice, relaxation). The intervention types will become the nodes in the NMA.

Assessment of frailty

We anticipate that a range of validated instruments (e.g., phenotype model; FRAIL scale; gait speed) and operationalised measures (e.g., use of walking aid; housebound) will be used to identify pre-frailty and frailty in included trial populations. We will report methods used for each trial, including cutpoints for identification of pre-frailty and frailty.

If a validated or operationalised frailty instrument has not been used, two reviewers with extensive clinical academic frailty expertise (AC/JG) will independently use the well-validated phenotype model as a framework to categorise study-level frailty profile (not frail; pre-frailty; frailty) of trial participants.[22] The model is based on five characteristics (weight loss; exhaustion; low energy expenditure; slow gait speed; low grip strength). Evidence of ≥3 indicates frailty, 1-2 pre-frailty and 0 not frail. The study-level categorisation will be based on whether trial eligibility criteria and/or trial baseline characteristics identify the five characteristics that indicate the presence of phenotypic pre-frailty or frailty. We anticipate that trials may include participants across different frailty categories, so will include additional categories ('not frail and pre-frail', 'pre-frail and frail' and 'all'). Any disagreements will be resolved by consensus. Our main analysis of the impact of frailty will only include trials that used a validated measure. Trials that used an operationalised measure or where study-level identification of frailty level is on the basis of eligibility criteria and baseline characteristics will be examined in sensitivity analyses.

Intention to treat and missing data

If both per-protocol and intention-to-treat analyses are reported for a trial, we will prioritise intention-to-treat data.[23] In all instances, we will report whether analysis was conducted on data that were

For peer review only - http://bmjopen.bmj.com/site/about/guidelines.xhtml

complete, complete after imputation or incomplete, and we will examine and report any material differences in results across these types. When results for main outcomes are missing for a trial, we will contact authors to request the missing data.

Risk of bias within individual studies

Researchers will independently assess the risk of bias (RoB) of each result of interest from each included trial, using Cochrane's RoB 2 - a revised tool for assessing risk of bias in randomised trials.[23] For cRCTs, we will additionally assess identification/recruitment bias, and the other issues such as loss of clusters, detailed in version 6 of the Cochrane Handbook.[24] For each domain in the RoB 2 tool, a judgement of high risk of bias, low risk of bias, or some concerns will be made, then an overall risk-of-bias judgement will be reached for each assessed outcome, with any disagreements resolved by consensus.

Summary measures

For each trial and each outcome separately, effect estimates and confidence intervals (CI) will be extracted comparing intervention and control groups. For continuous outcomes, we aim to extract the intervention effect as mean differences. We will consider using standardised mean difference if different measures are used for similar constructs. For binary outcomes, we will calculate risk ratios (RR) and odds ratios (OR). For survival (time-to-event) outcomes, hazard (rate) ratios will be extracted. Any details about non-proportional hazards will also be extracted. Outcomes at all timepoints will be recorded and grouped appropriately (around 6 months, around 12 months, and around 24 months). Where effect estimates and/or confidence intervals are not available, we will use other information (e.g., p-values, means for each group at follow-up, etc) to derive the information indirectly.

Unit of analysis issues

We will apply adjustment for trials that use cluster randomisation without adjusting standard errors.[24] As intra-class correlations needed to make such correction are rarely reported, we will use values obtained from external literature for the outcome examined (or if these are not available, use a single plausible value, and examine the impact of varying this value in sensitivity analysis).

Examination of potential effect modifiers

Treatment effect modifiers relate to methodological or clinical characteristics of the trials that influence the magnitude of treatment effects (on a given scale), and may include follow-up length, outcome definitions, trial quality (risk of bias), analysis and reporting standards (including risk of selective reporting), and the participant-level characteristics (e.g., leading to trial differences in

For peer review only - http://bmjopen.bmj.com/site/about/guidelines.xhtml

case-mix variation including frailty). When such effect modifiers are systematically different in trials making the same comparison(s), this manifests itself as between-study heterogeneity in treatment effects. When such effect modifiers are systematically different in the subsets of trials providing direct and indirect evidence about a particular comparison, this causes inconsistency (i.e., disagreement between the direct and indirect evidence for that comparison) in the NMA.

Hence, before any analysis, the distribution of potential effect modifiers will be examined across the studies to inform inconsistency concerns (whether direct and indirect evidence in the NMA are likely to coherent) and whether some trials should be removed to improve consistency. Clearly, such decisions will also be based on the inclusion and exclusion criteria for the project.

Data synthesis

We will meta-analyse the extracted effect estimates using modules within R and Stata, such as *metafor*, *metan*, *mvmeta* and *network*. Random-effects meta-analyses will be conducted, to allow for potential between-study heterogeneity in each intervention effect.[25] Restricted maximum likelihood (REML) estimation will be used to fit all the models, with 95% confidence intervals (CIs) derived using an approach to account for uncertainty in the estimate of heterogeneity (tau-squared), such as the Hartung-Knapp Sidik-Jonkman approach.[26] Initially for each outcome, we will perform a separate meta-analysis for each type of intervention, to provide summary effectiveness results based only on direct evidence. We will summarise odds ratios (ORs) and risk ratios (RRs) for binary outcomes, pooled (standardised) mean differences for continuous outcomes, and pooled hazard ratios (HRs) for survival outcomes. We will display forest plots, with study-specific estimates, confidence intervals and weights, alongside the summary (pooled) meta-analysis estimates, 95% CI, and (if appropriate) a 95% prediction interval.

Network meta-analysis (NMA)

An NMA will then be conducted (for each outcome separately), using a multivariate random-effects meta-analysis framework via the *network* module in Stata and using REML estimation (with confidence intervals derived accounting for uncertainty of variance estimates).[27] Nodes in the network will correspond to each intervention group as outlined previously. The NMA framework allows both direct and indirect evidence to contribute toward each intervention effect (treatment contrast), via a consistency assumption.[28] The within-study correlation of multiple intervention effects from the same trial (i.e., in multigroup trials) will be accounted for, and a common between-study variance assumed for all treatment contrasts in the network (thus implying a +0.5 between-study correlation for each pair of treatment effects). If possible, sensitivity to relaxing this assumption will be examined using model fit statistics. We will produce summary (pooled) effect estimates for each pair of treatments in the network, with 95% CI, and the borrowing of strength

For peer review only - http://bmjopen.bmj.com/site/about/guidelines.xhtml

statistic to reveal the contributions of indirect evidence. For binary outcomes, if possible we will do an NMA of both OR and RR, to check the robustness of conclusions to the choice of effect measure. Based on the results, the ranking of intervention types will be calculated using resampling methods, and quantified by the probabilities of being ranked first, second, ..., last, together with the mean rank and the Surface Under the Cumulative RAnking curve (SUCRA), with appropriate plots will be presented.

Assessment of inconsistency

The consistency assumption will be examined for each treatment comparison where there is direct and indirect evidence (seen as a closed loop within the network plot). This involves estimating direct and indirect evidence, and comparing the two.[29-31] The consistency assumption will also be examined across the whole network using 'design-by-treatment interaction' models, which allow an overall significance test for inconsistency. If evidence of inconsistency is found, explanations will be sought and resolved (e.g., with consideration of the distribution of effect modifiers; see earlier).

Examination of small-study effects

If there are 10 or more studies in a meta-analysis, funnel plots will be presented to examine small-study effects (potential publication bias). Egger's, Peter's and Debray's test of asymmetry will be used for continuous, binary and survival outcomes, respectively.

Examination of frailty impact

Meta-analysis (MA) results will initially be presented for all levels combined, then for frailty/pre-frailty where reported data permit. That is, a separate MA and NMA for each frailty type will be conducted. Indeed, this may also reduce any inconsistency (see above). We will also consider extending the MA and NMA models to a meta-regression, with frailty/pre-frailty as a study-level categorical covariate allowing effects of frailty/pre-frailty to vary for each treatment effect, to quantify if intervention effects vary according to population-level frailty.

All analyses to examine frailty impact will initially be restricted to trials using a validated measure. Sensitivity analyses will 1) be restricted to trials using the phenotype model to identify pre-frailty/frailty as an internationally-established reference standard, 2) include trials that used either a validated or an operationalised measure of frailty, 3) include all trials, including by study-level categorisation of frailty status.

Additional analyses

For peer review only - http://bmjopen.bmj.com/site/about/guidelines.xhtml

We will also run additional sensitivity analyses to present results of more recent evaluations, restricted to trials in the last 15 years. Meta-regression will be used to quantify differences in summary effects between studies at low risk of bias and other studies, and between those with shorter and longer lengths of follow-up. A multivariate network meta-analysis will be considered to accommodate all outcomes simultaneously, to examine if conclusions remain the same after accounting for the correlation amongst outcomes.[32] As mentioned, we will consider how relaxing the assumption of common between-study variances improves model fit.

Confidence in cumulative evidence

We will use the Grading of Recommendations Assessment, Development and Evaluation (GRADE) framework, adapted for NMA, to rate evidence quality.[33-36] Our assessment of quality of treatment effects will enable generation of GRADE evidence profiles for our individual intervention groupings for each outcome separately.

The assessment of quality of treatment effects will include presentation and rating of the quality of direct and indirect treatment estimates separately and combined in NMA[35], with a focus on first order loops for assessment of indirect treatment estimates. As we will include RCTs and cRCTs the starting point will be a high-quality evidence rating. For each estimate of treatment effect, we will assess risk of bias, inconsistency, indirectness, imprecision and publication bias. We will make an overall judgement on whether the quality rating for each effect warrants downgrading on the basis of limitations in each of the domains, aligned with GRADE guidance[36]. We will not consider imprecision when rating the direct and indirect estimates to inform the combined NMA rating, aligned with recent guidance.[33] Furthermore, in the presence of incoherence between direct and indirect estimates, we will assess the certainty of evidence of each estimate to guide whether or not the network estimate is downgraded.[33]

Patient and public involvement

Our established patient and public involvement Frailty Oversight Group (FOG) provides connections to the whole spectrum of older people, with a focus on those living with frailty. We have consulted our FOG throughout the development of this protocol and discussed plans in detail at quarterly meetings. A specific example of their influence is our selection of main and additional outcomes of importance for older people. We plan to involve FOG in our intervention grouping and dissemination of this review.

Timelines

For peer review only - http://bmjopen.bmj.com/site/about/guidelines.xhtml

Formal screening of search results began in January 2020. Data extraction began in May 2020. Risk of bias assessment and data extraction have not yet begun. We are currently updating our searches (September 2020). The project is due to complete in October 2021.

Ethics and dissemination

Ethics approval is not needed as this systematic review will use aggregated, anonymised data that is available in the public domain.

This will be the first systematic review with network meta-analysis comparing the effectiveness of community-based complex interventions to sustain independence for older people, including the effect of frailty and pre-frailty. The review will use a detailed analysis to group the included interventions to identify the best configurations. Further, it will also review the quality of evidence using the GRADE approach.[35] We will disseminate the findings widely through communication with healthcare providers, conference presentations and academic publications. We will adhere to PRISMA-NMA reporting guidelines.[14] Hence, this systematic review will produce transparent and accessible results that are of great relevance and applicability for a wide audience including policymakers, commissioners, health/social care professionals, older people and researchers working with an older population.

Acknowledgements

We are grateful to our Information Specialist, Deirdre Andre, for her assistance developing the search strategy.

Footnotes

Authors' contributions: AC and TFC were responsible for conception and design of the study. RDR, JE, and RB designed the statistical analysis plan. NL, RB, MJ, EP, RR, AF, JRFG provided critical revisions of all aspects of the review. AC and TFC are guarantor of the review. All authors have reviewed and approved the final manuscript.

Funding: This project is funded by the National Institute for Health Research (NIHR) Health Technology Assessment programme (project reference NIHR128862). The views expressed are those of the author(s) and not necessarily those of the NIHR or the Department of Health and Social Care. AF receives funding from an NIHR Senior Investigator award.

Disclaimer: The funders had no role in the design of the planned study or preparation of this protocol.

Competing interests: The authors declare that they have no competing interests.

Abbreviations: CGA: comprehensive geriatric assessment; GRADE: Grading of Recommendations, Assessment, Development and Evaluation; MA: Meta-analysis; NIHR: National Institute of Health Research; NHS: National Health Service; NMA: Network meta-analysis; PICOS: Population, intervention, comparator, outcomes, study design; PRISMA-P: Preferred Reporting Items for Systematic review and Meta-Analysis Protocols; RoB: Risk of bias; SUCRA: Surface Under the Cumulative RAnking curve; TIDieR: Template for Intervention Description and Replication

For peer review only - http://bmjopen.bmj.com/site/about/guidelines.xhtml

References

1. United Nations. Department of Economic and Social Affairs, Population Division. World Population Prospects 2019. Secondary United Nations. Department of Economic and Social Affairs, Population Division. World Population Prospects 2019. https://population.un.org/wpp/.
2. Overview of the UK population: November 2018. Office for National Statistics, London, UK.
3. Government of United Kingdom. The Grand Challenge missions: Ageing society. Secondary Government of United Kingdom. The Grand Challenge missions: Ageing society 13/09/2019 2019. https://www.gov.uk/government/publications/industrial-strategy-the-grand-challenges/missions.
4. World Health Organization. Decade of Healthy Ageing: 2020-2030. Secondary World Health Organization. Decade of Healthy Ageing: 2020-2030. https://www.who.int/ageing/decade-of-healthy-ageing.
5. Gabriel Z, Bowling ANN. Quality of life from the perspectives of older people. *Ageing and Society* 2004;**24**(5):675-91.
6. Clegg A, Young J, Iliffe S, et al. Frailty in elderly people. Lancet 2013;**381**(9868):752-62.
7. Collard RM, Boter H, Schoevers RA, et al. Prevalence of frailty in community-dwelling older persons: a systematic review. *J Am Geriatr Soc* 2012;**60**(8):1487-92.
8. UK health and social care spending, 2016. Office for Budget Responsibility. London, UK.
9. Care for older people. Projected expenditure to 2022 on social care and continuing health care for England's older population. Nuffield Trust 2012. London, UK.
10. Han L, Clegg A, Doran T, et al. The impact of frailty on healthcare resource use: a longitudinal analysis using the Clinical Practice Research Datalink in England. *Age Ageing* 2019;**48**(5):665-71.
11. Beswick AD, Rees K, Dieppe P, et al. Complex interventions to improve physical function and maintain independent living in elderly people: a systematic review and meta-analysis. *Lancet* 2008;**371**(9614):725-35.
12. Stuck AE, Egger M, Hammer A, et al. Home visits to prevent nursing home admission and functional decline in elderly people: systematic review and meta-regression analysis. *JAMA* 2002;**287**(8):1022-8.
13. Tappenden P, Campbell F, Rawdin A, et al. The clinical effectiveness and cost-effectiveness of home-based, nurse-led health promotion for older people: a systematic review. *Health Technol Assess* 2012;**16**(20):1-72.
14. Hutton B, Salanti G, Caldwell DM, et al. The PRISMA Extension Statement for Reporting of Systematic Reviews Incorporating Network Meta-analyses of Health Care Interventions: Checklist and Explanations. *Annals of Internal Medicine* 2015;**162**(11):777-84.
15. Moher D, Shamseer L, Clarke M, et al. Preferred reporting items for systematic review and meta-analysis protocols (PRISMA-P) 2015 statement. *Systematic Reviews* 2015;**4**(1):1.

For peer review only - http://bmjopen.bmj.com/site/about/guidelines.xhtml

16. Crocker T, Forster A, Young J, et al. Physical rehabilitation for older people in long-term care. *Cochrane Database Syst Rev* 2013(2):CD004294.
17. Low L-F, Fletcher J, Goodenough B, et al. A Systematic Review of Interventions to Change Staff Care Practices in Order to Improve Resident Outcomes in Nursing Homes. *PLOS ONE* 2015;**10**(11):e0140711.
18. Tricco AC, Thomas SM, Veroniki AA, et al. Comparisons of Interventions for Preventing Falls in Older Adults: A Systematic Review and Meta-analysis. *JAMA* 2017;**318**(17):1687-99.
19. GRADEpro GDT: GRADEpro Guideline Development Tool [Software]. McMaster University, 2020 (developed by Evidence Prime, Inc.). Available from gradepro.org.
20. Hoffmann TC, Glasziou PP, Boutron I, et al. Better reporting of interventions: template for intervention description and replication (TIDieR) checklist and guide. *BMJ* 2014;**348**:g1687.
21. Patton M. *Qualitative research and evaluation methods: integrating theory and practice. 4th edition. SAGE publishing, Los Angeles*, 2015.
22. Fried LP, Tangen CM, Walston J, et al. Frailty in older adults: evidence for a phenotype. *J Gerontol A Biol Sci Med Sci* 2001;**56**(3):M146-56.
23. Sterne JAC, Savović J, Page MJ, et al. RoB 2: a revised tool for assessing risk of bias in randomised trials. *BMJ* 2019;**366**:l4898.
24. Higgins JPT, Thomas J, Chandler J, et al., editors. *Cochrane Handbook for Systematic Reviews of Interventions*. 2nd ed. Chichester, UK: Cochrane Collaboration and John Wiley & Sons, 2019.
25. Riley RD, Higgins JP, Deeks JJ. Interpretation of random effects meta-analyses. *BMJ* 2011;**342**:d549.
26. Langan D, Higgins JPT, Jackson D, et al. A comparison of heterogeneity variance estimators in simulated random-effects meta-analyses. *Res Synth Methods* 2018.
27. Riley RD, Jackson D, Salanti G, et al. Multivariate and network meta-analysis of multiple outcomes and multiple treatments: rationale, concepts, and examples. *BMJ* 2017;**358**:j3932.
28. Dias S, Ades AE, Welton NJ, et al. *Network meta-analysis for decision-making*: John Wiley & Sons, 2018.
29. Dias S, Welton NJ, Caldwell DM, et al. Checking consistency in mixed treatment comparison meta-analysis. *Stat Med* 2010;**29**(7-8):932-44.
30. Dias S, Welton NJ, Sutton AJ, et al. Evidence synthesis for decision making 4: inconsistency in networks of evidence based on randomized controlled trials. *Med Decis Making* 2013;**33**(5):641-56.
31. Veroniki AA, Vasiliadis HS, Higgins JP, et al. Evaluation of inconsistency in networks of interventions. *Int J Epidemiol* 2013;**42**(1):332-45.
32. Jackson D, Bujkiewicz S, Law M, et al. A matrix-based method of moments for fitting multivariate network meta-analysis models with multiple outcomes and random inconsistency effects. *Biometrics* 2018;**74**(2):548-56.
33. Brignardello-Petersen R, Bonner A, Alexander PE, et al. Advances in the GRADE approach to rate the certainty in estimates from a network meta-analysis. *J Clin Epidemiol* 2018;**93**:36-44.

For peer review only - http://bmjopen.bmj.com/site/about/guidelines.xhtml

34. Guyatt GH, Oxman AD, Schunemann HJ, et al. GRADE guidelines: a new series of articles in the Journal of Clinical Epidemiology. *J Clin Epidemiol* 2011;**64**(4):380-2.
35. Puhan MA, Schunemann HJ, Murad MH, et al. A GRADE Working Group approach for rating the quality of treatment effect estimates from network meta-analysis. *BMJ* 2014;**349**:g5630.
36. Schünemann H, Brożek J, Guyatt G, et al. *Handbook for grading the quality of evidence and the strength of recommendations using the GRADE approach. Available from https://gdt.gradepro.org/app/handbook/handbook.html*, 2013.

For peer review only

For peer review only - http://bmjopen.bmj.com/site/about/guidelines.xhtml

Appendix A: MEDLINE search strategy

Database: Ovid MEDLINE(R) and Epub Ahead of Print, In-Process & Other Non-Indexed Citations and Daily <1946 to December 31, 2019>

Search Strategy:

----

1 randomized controlled trial.pt. (497917)

2 controlled clinical trial.pt. (93495)

3 randomized.ab. (465667)

4 placebo.ab. (204054)

5 clinical trials as topic.sh. (189709)

6 randomly.ab. (324606)

7 trial.ti. (210688)

8 or/1-7 (1260622)

9 exp animals/ not humans.sh. (4659772)

10 8 not 9 [Cochrane Highly Sensitive Search Strategy for identifying randomized trials in MEDLINE: sensitivity- and precision-maximizing version (2008 revision] (1159627)

11 Clinical Trial, Phase III/ (16136)

12 ("phase 3" or "phase3" or "phase III" or "P3" or "PIII").ti,ab,kw. (64901)

13 11 or 12 [search filter for phase three trials to suppplement Cochrane HSSS, Cooper 2019] (70728)

14 10 or 13 [final RCT filter] (1199211)

15 (frail* or prefrailty).tw. (19992)

16 exp aged/ (3036862)

17 geriatrics/ (29616)

18 (elder* or older or old people* or old person* or old wom#n*1 or old m#n*1 or old male*1 or old female*1 or old adult*1 or old age* or aging or ageing or geriatric* or senior citizen* or seniors or pensioner* or veteran* or sexagenarian* or septuagenarian* or octogenarian* or nonagenarian* or centenarian*).tw,kf. (1233323)

19 (over adj2 ("60" or "61" or "62" or "63" or "64" or "65" or "66" or "67" or "68" or "69" or "70" or "71" or "72" or "73" or "74" or "75" or "76" or "77" or "78" or "79" or "80" or "81" or "82" or "83" or "84" or "85" or "86" or "87" or "88" or "89" or "90" or "91" or "92" or "93" or "94" or "95" or "96" or "97" or "98" or "99" or "100") adj years).tw. (19080)

20 or/15-19 [older or frail people] (3792854)

21 independent living/ (5105)

22 community health services/ (31116)

23 community health nursing/ (19479)

24 Community support services.tw. (163)

25 exp managed care programs/ (39897)

For peer review only

26 (health maintenance organi?ation* or HMO*).tw. (13072)

27 (Social adj3 services).tw. (9360)

28 Voluntary services.tw. (93)

29 *home nursing/ (5264)

30 House Calls/ (3475)

31 house call*.tw. (615)

32 (home adj5 visit*).tw. (11016)

33 ((general practice or primary care or nurse* or group or ambulatory clinic or geriatric clinic) adj3 visit*).tw. (8476)

34 *geriatric assessment/ (12510)

35 (pharmac* adj2 visit).tw. (182)

36 ((home or house) adj2 appointment*).tw. (42)

37 Home Care Services/ (32888)

38 Home care service*.tw. (1725)

39 *health services for the aged/ (13598)

40 home health nursing/ (319)

41 district nursing.tw. (649)

42 health visit*.ti. or health visit*.ab. /freq=2 (2224)

43 community matron*.ti. or community matron*.ab. /freq=2 (83)

44 (home adj3 (intervention* or support* or assessment*)).tw. (7580)

45 preventive health services/ (13212)

46 ((preventive* or preventative*) adj5 medicine).tw. (6792)

47 preventative medicine/ (11603)

48 ((preventive* or preventative*) adj3 (program* or intervent* or support* or care or service* or approach* or case management or measure* or OT or occupational therapy or assess*)).tw. (57977)

49 or/21-48 [specific interventions] (261556)

50 geriatric nursing/ (13511)

51 geriatric nurs*.tw,kf. (1086)

52 or/50-51 [geriatric nursing] (13877)

53 community.ti,ab,kf. (464179)

54 community health services/ or community health nursing/ or community mental health services/ or community pharmacy services/ (71636)

55 "domiciliary care"/ (32888)

56 aftercare/ (8799)

57 primary health care/ (75131)

For peer review only

58 (domiciliary or (social support and home*) or ((homecare or medical) adj2 home) or (home and package*) or (outreach and home) or (alternative setting and home) or home visit* or home manag* or homecare or home care or home therap* or (model* adj1 home*) or home program* or home monitor*).tw. (53213)

59 ((live or living or lived or dwell*) adj5 ("at home" or "own home" or "in home" or alone or independent*)).tw. (15304)

60 (home-based or homebased or homebound).tw. (10615)

61 (Home care or primary care or primary health care or primary healthcare).tw. (146114)

62 or/53-61 [interventions in a community or home setting] (709468)

63 52 and 62 [geriatric nursing and interventions in a community or home setting] (1982)

64 49 or 63 [all interventions] (262247)

65 (coronary heart disease or CHD or chronic obstructive pulmonary disease or COPD or kidney failure or CKD or Heart failure or diabetes or asthma or cancer or schizophrenia or severe mental illness*).ti. (1420118)

66 64 not 65 [all intervenions excluding specific diseases in title] (247185)

67 14 and 20 and 66 [RCTS and older people and interventions] (6170)

For peer review only

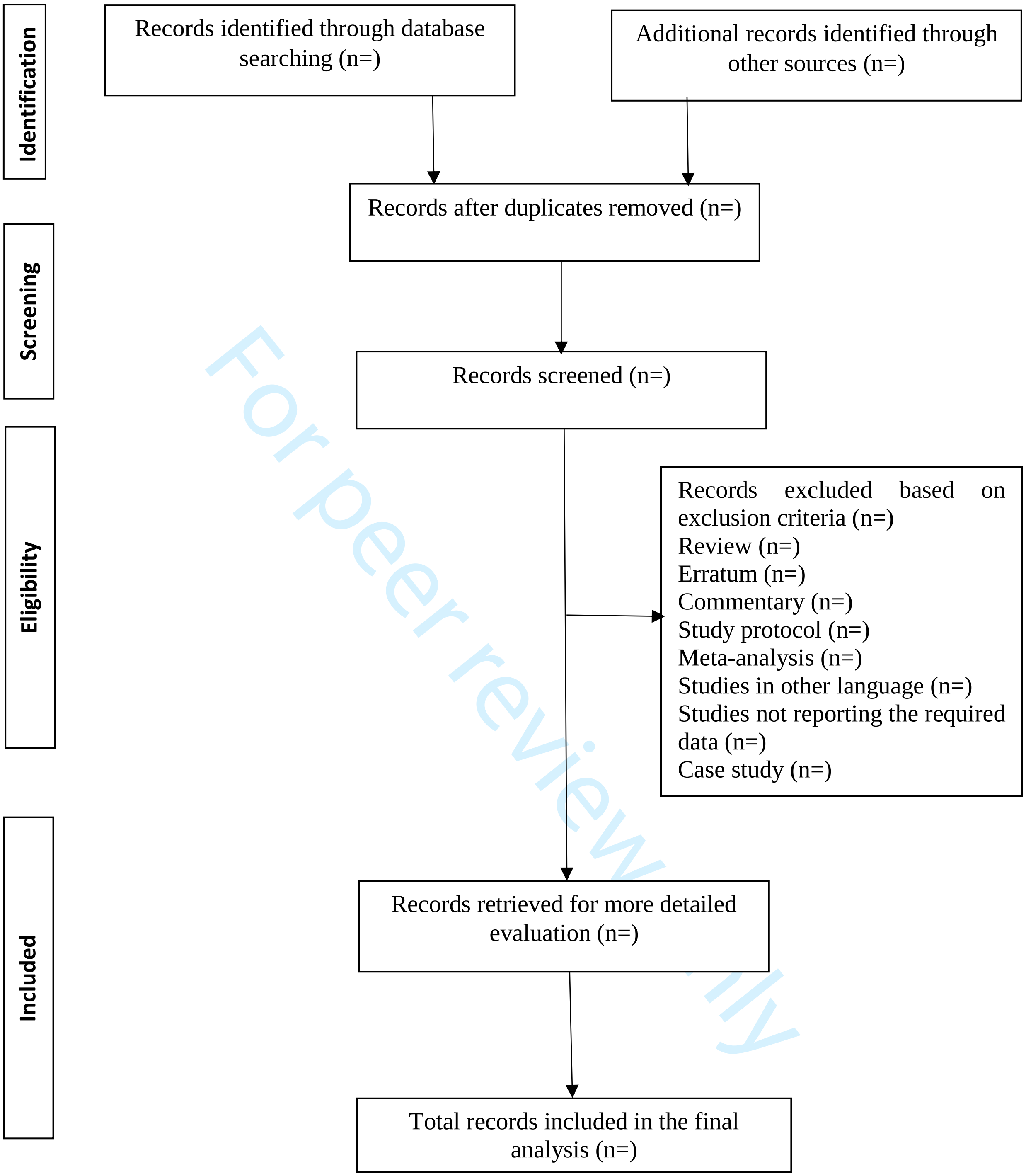

N, number.

Supplementary figure S1

For peer review only

Additional file 1: PRISMA-P checklist

PRISMA-P (Preferred Reporting Items for Systematic review and Meta-Analysis Protocols) 2015 checklist: recommended items to address in a systematic review protocol*

Section and topic	Item No	Checklist item	(Page No.#)
**ADMINISTRATIVE INFORMATION**			
Title:			
Identification	1a	Identify the report as a protocol of a systematic review	1
Update	1b	If the protocol is for an update of a previous systematic review, identify as such	NA
Registration	2	If registered, provide the name of the registry (such as PROSPERO) and registration number	3
Authors:			
Contact	3a	Provide name, institutional affiliation, e-mail address of all protocol authors; provide physical mailing address of corresponding author	1-2
Contributions	3b	Describe contributions of protocol authors and identify the guarantor of the review	15
Amendments	4	If the protocol represents an amendment of a previously completed or published protocol, identify as such and list changes; otherwise, state plan for documenting important protocol amendments	NA
Support:			
Sources	5a	Indicate sources of financial or other support for the review	15
Sponsor	5b	Provide name for the review funder and/or sponsor	
Role of sponsor or funder	5c	Describe roles of funder(s), sponsor(s), and/or institution(s), if any, in developing the protocol	
**INTRODUCTION**			
Rationale	6	Describe the rationale for the review in the context of what is already known	5-6
Objectives	7	Provide an explicit statement of the question(s) the review will address with reference to participants, interventions, comparators, and outcomes (PICO)	6
**METHODS**			
Eligibility criteria	8	Specify the study characteristics (such as PICO, study design, setting, time frame) and report characteristics (such as years considered, language, publication status) to be used as criteria for eligibility for the review	7-9
Information sources	9	Describe all intended information sources (such as electronic databases, contact with study authors, trial registers or other grey literature sources) with planned dates of coverage	9

For peer review only

Search strategy	10	Present draft of search strategy to be used for at least one electronic database, including planned limits, such that it could be repeated	Appendix-A
Study records:			
Data management	11a	Describe the mechanism(s) that will be used to manage records and data throughout the review	9
Selection process	11b	State the process that will be used for selecting studies (such as two independent reviewers) through each phase of the review (that is, screening, eligibility and inclusion in meta-analysis)	9
Data collection process	11c	Describe planned method of extracting data from reports (such as piloting forms, done independently, in duplicate), any processes for obtaining and confirming data from investigators	9
Data items	12	List and define all variables for which data will be sought (such as PICO items, funding sources), any pre-planned data assumptions and simplifications	9
Outcomes and prioritization	13	List and define all outcomes for which data will be sought, including prioritization of main and additional outcomes, with rationale	8-9
Risk of bias in individual studies	14	Describe anticipated methods for assessing risk of bias of individual studies, including whether this will be done at the outcome or study level, or both; state how this information will be used in data synthesis	11-12
Data synthesis	15a	Describe criteria under which study data will be quantitatively synthesised	12-14
	15b	If data are appropriate for quantitative synthesis, describe planned summary measures, methods of handling data and methods of combining data from studies, including any planned exploration of consistency (such as I^2, Kendall's τ)	
	15c	Describe any proposed additional analyses (such as sensitivity or subgroup analyses, meta-regression)	
	15d	If quantitative synthesis is not appropriate, describe the type of summary planned	
Meta-bias(es)	16	Specify any planned assessment of meta-bias(es) (such as publication bias across studies, selective reporting within studies)	14
Confidence in cumulative evidence	17	Describe how the strength of the body of evidence will be assessed (such as GRADE)	14

*** It is strongly recommended that this checklist be read in conjunction with the PRISMA-P Explanation and Elaboration (cite when available) for important clarification on the items. Amendments to a review protocol should be tracked and dated. The copyright for PRISMA-P (including checklist) is held by the PRISMA-P Group and is distributed under a Creative Commons Attribution Licence 4.0.**

From: Shamseer L, Moher D, Clarke M, Ghersi D, Liberati A, Petticrew M, Shekelle P, Stewart L, PRISMA-P Group. Preferred reporting items for systematic review and meta-analysis protocols (PRISMA-P) 2015: elaboration and explanation. BMJ. 2015 Jan 2;349(jan02 1):g7647.

For peer review only

BMJ Open

Community-based complex interventions to sustain independence in older people, stratified by frailty: a protocol for a systematic review and network meta-analysis

Journal:	*BMJ Open*
Manuscript ID	bmjopen-2020-045637.R1
Article Type:	Protocol
Date Submitted by the Author:	21-Dec-2020
Complete List of Authors:	Crocker, Thomas; Bradford Teaching Hospitals NHS Foundation Trust, Academic Unit for Ageing and Stroke Research<br>Clegg, Andrew; University of Leeds, Academic Unit for Ageing and Stroke Research, Bradford Institute for Health Research, Bradford Teaching Hospitals NHS Foundation Trust<br>Riley, Richard; Keele University, Centre for Prognosis Research, School of Medicine<br>Lam, Natalie; Bradford Teaching Hospitals NHS Foundation Trust, Bradford Institute for Health Research, Academic Unit for Ageing and Stroke Research<br>Bajpai, Ram; Keele University, School of Medicine; University of Leeds Leeds Institute of Health Sciences<br>Jordão, Magda; Bradford Teaching Hospitals NHS Foundation Trust, Bradford Institute for Health Research, Academic Unit for Ageing and Stroke Research<br>Patetsini, Eleftheria; Bradford Teaching Hospitals NHS Foundation Trust, Bradford Institute for Health Research, Academic Unit for Ageing and Stroke Research<br>Ramiz, Ridha; Bradford Teaching Hospitals NHS Foundation Trust, Bradford Institute for Health Research, Academic Unit for Ageing and Stroke Research<br>Ensor, Joie; Keele University, Centre for Prognosis Research, School of Medicine<br>Forster, Anne; University of Leeds, Academic Unit for Ageing and Stroke Research, Bradford Institute for Health Research, Bradford Teaching Hospitals NHS Foundation Trust<br>Gladman, John; University of Nottingham, Nottingham University Hospitals NHS Trust, NIHR Applied Research Collaboration (ARC) East Midlands, NIHR Nottingham Biomedical Research Centre
**Primary Subject Heading**:	Geriatric medicine
Secondary Subject Heading:	General practice / Family practice, Health services research, Occupational and environmental medicine, Rehabilitation medicine
Keywords:	GERIATRIC MEDICINE, Clinical trials < THERAPEUTICS, PRIMARY CARE, PREVENTIVE MEDICINE, REHABILITATION MEDICINE, OCCUPATIONAL & INDUSTRIAL MEDICINE

I, the Submitting Author has the right to grant and does grant on behalf of all authors of the Work (as defined in the below author licence), an exclusive licence and/or a non-exclusive licence for contributions from authors who are: i) UK Crown employees; ii) where BMJ has agreed a CC-BY licence shall apply, and/or iii) in accordance with the terms applicable for US Federal Government officers or employees acting as part of their official duties; on a worldwide, perpetual, irrevocable, royalty-free basis to BMJ Publishing Group Ltd ("BMJ") its licensees and where the relevant Journal is co-owned by BMJ to the co-owners of the Journal, to publish the Work in this journal and any other BMJ products and to exploit all rights, as set out in our licence.

The Submitting Author accepts and understands that any supply made under these terms is made by BMJ to the Submitting Author unless you are acting as an employee on behalf of your employer or a postgraduate student of an affiliated institution which is paying any applicable article publishing charge ("APC") for Open Access articles. Where the Submitting Author wishes to make the Work available on an Open Access basis (and intends to pay the relevant APC), the terms of reuse of such Open Access shall be governed by a Creative Commons licence – details of these licences and which Creative Commons licence will apply to this Work are set out in our licence referred to above.

Other than as permitted in any relevant BMJ Author's Self Archiving Policies, I confirm this Work has not been accepted for publication elsewhere, is not being considered for publication elsewhere and does not duplicate material already published. I confirm all authors consent to publication of this Work and authorise the granting of this licence.

For peer review only

Community-based complex interventions to sustain independence in older people, stratified by frailty: a protocol for a systematic review and network meta-analysis

Thomas F. Crocker, Andrew Clegg, Richard D. Riley, Natalie Lam, Ram Bajpai, Magda Jordão, Eleftheria Patetsini, Ridha Ramiz, Joie Ensor, Anne Forster, John R.F. Gladman

Thomas F. Crocker
1. Academic Unit for Ageing and Stroke Research, Bradford Institute for Health Research, Bradford Teaching Hospitals NHS Foundation Trust, Bradford, UK
   Email: tom.crocker@bthft.nhs.uk ; ORCID: 0000-0001-7450-3143

Andrew Clegg
1. Academic Unit for Ageing and Stroke Research, University of Leeds, Bradford Institute for Health Research, Bradford Teaching Hospitals NHS Foundation Trust, Bradford, UK
   Email: a.p.clegg@leeds.ac.uk ; ORCID: 0000-0001-5972-1097

Richard D. Riley
1. Centre for Prognosis Research, School of Medicine, Keele University, Keele, UK
   Email: r.riley@keele.ac.uk ; ORCID: 0000-0001-8699-0735

Natalie Lam
1. Academic Unit for Ageing and Stroke Research, Bradford Institute for Health Research, Bradford Teaching Hospitals NHS Foundation Trust, Bradford, UK
   Email: natalie.lam@bthft.nhs.uk ; ORCID: 0000-0001-8591-444X

Ram Bajpai
1. School of Medicine, Keele University, Keele, UK
2. Leeds Institute of Health Sciences, University of Leeds, Leeds, UK
   Email: r.bajpai@keele.ac.uk ; ORCID: 0000-0002-1227-2703

Magda Jordão
1. Academic Unit for Ageing and Stroke Research, Bradford Institute for Health Research, Bradford Teaching Hospitals NHS Foundation Trust, Bradford, UK
   Email: magda.jordao@bthft.nhs.uk ; ORCID: 0000-0003-2108-2677

Eleftheria Patetsini
1. Academic Unit for Ageing and Stroke Research, Bradford Institute for Health Research, Bradford Teaching Hospitals NHS Foundation Trust, Bradford, UK
   Email: eleftheria.patetsini@bthft.nhs.uk ; ORCID: 0000-0001-8393-982X

Ridha Ramiz
1. Academic Unit for Ageing and Stroke Research, Bradford Institute for Health Research, Bradford Teaching Hospitals NHS Foundation Trust, Bradford, UK
   Email: ridha.ramiz@bthft.nhs.uk ; ORCID: 0000-0002-3569-8731

Joie Ensor
1. Centre for Prognosis Research, School of Medicine, Keele University, Keele, UK
   Email: j.ensor@keele.ac.uk ; ORCID: 0000-0001-7481-0282

Anne Forster
1. Academic Unit for Ageing and Stroke Research, University of Leeds, Bradford Institute for Health Research, Bradford Teaching Hospitals NHS Foundation Trust, Bradford, UK
   Email: a.forster@leeds.ac.uk ; ORCID: 0000-0001-7466-4414

John R.F. Gladman

For peer review only - http://bmjopen.bmj.com/site/about/guidelines.xhtml

1. University of Nottingham, Nottingham, UK
2. Nottingham University Hospitals NHS Trust, Nottingham, UK
3. NIHR Applied Research Collaboration (ARC) East Midlands, UK
4. NIHR Nottingham Biomedical Research Centre, UK
   Email: john.gladman@nottingham.ac.uk ; ORCID: 0000-0002-8506-7786

Corresponding author:

Thomas F. Crocker
Academic Unit for Ageing and Stroke Research, Bradford Institute for Health Research, Bradford Teaching Hospitals NHS Foundation Trust, Bradford, UK
Email: tom.crocker@bthft.nhs.uk ; ORCID: 0000-0001-7450-3143

Word counts:

Abstract: 300

Manuscript: 4155

For peer review only - http://bmjopen.bmj.com/site/about/guidelines.xhtml

Abstract

Introduction

Maintaining independence is a primary goal of community health and care services for older people but there is currently insufficient guidance about which services to implement. Therefore, we aim to synthesise evidence on the effectiveness of community-based complex interventions to sustain independence for older people, including the effect of frailty, and group interventions to identify the best configurations.

Methods and analysis

Systematic review and network meta-analysis (NMA). We will include randomised controlled trials (RCTs) and cluster RCTs of community-based complex interventions to sustain independence for older people living at home (mean age ≥ 65 years), compared with usual care or another complex intervention. We will search MEDLINE (1946-), Embase (1947-), CINAHL (1981-), PsycINFO (1806-), CENTRAL and clinical trial registries from inception to September 2020, without date/language restrictions, and scan included papers' reference lists. Main outcomes: living at home, activities of daily living (basic/instrumental), home-care services usage, hospitalisation, care home admission, costs and cost-effectiveness. Additional outcomes: health status, depression, loneliness, falls and mortality. Interventions will be coded, summarised and grouped. An NMA using a multivariate random-effects model for each outcome separately will determine the relative effects of different complex interventions. For each outcome, we will produce summary effect estimates for each pair of treatments in the network, with 95% confidence interval, ranking plots and measures, and the borrowing of strength statistic. Inconsistency will be examined using a 'design-by-treatment interaction' model. We will assess risk of bias (Cochrane tool, version 2) and certainty of evidence using the Grading of Recommendations Assessment, Development and Evaluation for NMA approach.

Ethics and dissemination

This research will use aggregated, anonymised, published data. Findings will be reported according to Preferred Reporting Items for Systematic Reviews and Meta-analyses guidance. They will be disseminated to policymakers, commissioners, and providers, and via conferences and scientific journals.

PROSPERO registration: CRD42019162195

Keywords: primary health care practice, rehabilitation therapy, reablement, comprehensive geriatric assessment, preventive health services, multicomponent package of care, community dwelling person, frail elderly, ageing well in place, resilience.

For peer review only - http://bmjopen.bmj.com/site/about/guidelines.xhtml

Strengths and limitations of this study

- This will be the first systematic review with network meta-analysis comparing the effectiveness of community-based complex interventions to sustain independence for older people, including the effect of frailty and pre-frailty.
- A careful process to group interventions, including summarising each intervention with the Template for Intervention Description and Replication (TIDieR), will produce an analysis that is transparent and relevant to policymakers, commissioners and providers.
- In addition to the direct treatment effects, indirect treatment effects will be analysed using a random-effects network meta-analysis allowing us to compare different service models with each other.
- Summary of Findings tables developed using the GRADE approach for NMA will provide an accessible assessment of the certainty and size of treatment effects.
- The review is likely to be limited by lack of detail about the experimental conditions and wider care system in some trials, and the lack of consistent outcome measures.

For peer review only

For peer review only - http://bmjopen.bmj.com/site/about/guidelines.xhtml

Introduction

Global population projections indicate that older people are the fastest growing demographic, with the percentage of people aged 65 and over expected to almost double by 2050[1], and similar projections for developed countries such as the UK.[2] Given this success of increasing lifespan, current policy and initiatives such as the World Health Organization's Decade of Healthy Ageing emphasise increasing the number of years lived *in good health.*[3 4] This focus on sustaining health is crucial for enabling people to realise their strong preference for living with independence within a community[5]. Additionally, older people are core users of health and care services, so the ageing population demographic has profound implications for service planning and delivery. However, there is currently insufficient guidance for policymakers, commissioners and providers about which community services should be implemented.

Frailty is an especially problematic feature of population ageing, with increased risk of losing independence, hospitalisation, care home admission and mortality.[6] In the UK, around 10% of people aged 65 and over have frailty, rising to around 50% of people aged over 85.[7] UK NHS expenditure increases considerably with advancing age, with a threefold increase for people aged over 70.[8] UK social care expenditure for older people is expected to rise to £12.7 billion by 2022.[9] Extra annual cost to the healthcare system per person was £561.05 for mild, £1,208.60 for moderate and £2,108.20 for severe frailty with reference to 2013/14 UK costs.[10] This estimates a total additional cost of £5.8 billion per year across the UK.[10] These findings are mirrored in other developed countries.

There is a critical evidence gap regarding which community-based interventions are clinically and cost-effective, and therefore appropriate, for older people, including those living with frailty and pre-frailty. This evidence gap means that there is considerable uncertainty regarding how interventions should best be configured and commissioned. Previous systematic reviews and meta-analyses have reported evidence for clinical and cost-effectiveness of community-based complex interventions for reducing hospital admission, nursing home admission, falls and functional decline in older people.[11-13] However, previous reviews have not used NMA to summarise whether different types of interventions have differential effects on outcomes, limiting usefulness for policymakers, health and social care commissioners and providers. Moreover, few systematic reviews have investigated such interventions delivered outside of the home environment. A landmark 2008 systematic review and meta-analysis summarised evidence from 89 trials including 97,984 people.[11] The review reported that, in general, complex interventions provided in the community are effective for older people but lacked detail about what types of complex care improve outcomes, and does not include studies published over the last decade, which are potentially influential. This review only considered frailty in relation to one intervention (Comprehensive Geriatric Assessment)

For peer review only - http://bmjopen.bmj.com/site/about/guidelines.xhtml

and used a disability-based, non-validated definition of frailty to categorise trials. Standard meta-analysis techniques were used to synthesise the evidence.

Recognising that research evidence, understanding of frailty, and meta-analysis methods have advanced considerably in the last decade the review requires a contemporary update to identify how interventions might best be configured to improve outcomes and inform commissioning and delivery of evidence-based services.

Specific review questions are:

(i) Do community-based complex interventions to sustain independence in older people increase living at home, independence and health-related quality of life?
(ii) Do community-based complex interventions to sustain independence in older people reduce home care requirement, depression, loneliness, falls, hospitalisation, care home admission, costs and mortality?
(iii) How should interventions be grouped for network meta-analysis (NMA)?
(iv) What is the optimal configuration of community-based complex interventions to sustain independence in older people?
(v) Do intervention effects differ by frailty level (not frail; pre-frailty; frailty)?

Objectives

The overall aim of this systematic review is to synthesise evidence on the effectiveness of community-based complex interventions to sustain independence in older people, including the effect of frailty and pre-frailty, and group interventions to identify the best configurations. For this systematic review we define sustaining independence to mean maintaining or improving independence in activities of daily living (washing, dressing, grooming, toileting, walking, preparing meals, doing housework, managing finances, assisting others, etc), but not only one of these specific activities (e.g., walking only). The specific objectives are as follows:

(i) To identify randomised controlled trials (RCTs) and cluster randomised controlled trials (cRCTs) of community-based complex interventions to sustain independence in older people.
(ii) To synthesise evidence of their effectiveness for key outcomes in a meta-analysis of study-level data.
(iii) To identify key intervention components and study-level frailty to inform groupings for NMA and meta-regression.
(iv) To compare effectiveness of different intervention configurations using NMA.
(v) To investigate the impact of frailty and pre-frailty using meta-regression.

For peer review only - http://bmjopen.bmj.com/site/about/guidelines.xhtml

Methods and analysis

This protocol is registered on the International Prospective Register of Systematic Reviews (PROSPERO) (CRD42019162195). This protocol is reported in accordance with the reporting guidance provided in the Preferred Reporting Items for Systematic Reviews and Meta-Analyses Protocols (PRISMA-P) statement and PRISMA-NMA reporting guidelines.[14 15]

Eligibility criteria

We will select studies according to their design and the PICO criteria: participants, intervention, comparator, and outcome(s) of interest.

Study design: randomised controlled trials (RCTs) and cluster randomised controlled trials (cRCTs). Where only one unit of randomisation (an individual or cluster) is allocated to an arm of a trial, we will exclude the trial as the treatment effect is completely confounded with the unit. We accept minimisation as a method of sequence generation, in keeping with the Cochrane risk of bias guidance. Crossover trials are also eligible; however, we will only use outcome data from the pre-crossover period.

Participants (population): older people living at home (mean age of participants 65 years or older). We will exclude trials of residents of care/nursing homes as these are the subject of other large-scale reviews.[16 17] If not all participants are living at home, we will only include the trial if data can be extracted specifically for these participants.

Intervention: Aligned with our focus on community-based complex interventions, trials will be considered eligible if:

- the intervention is both initiated and mainly provided in the community;
- the intervention includes two or more interacting components (intervention practices, structural elements, and contextual factors);
- the intervention is targeted at the individual person, with provision of appropriate specialist care; and
- a focus of the intervention is sustaining (maintaining or improving) the person's independence.

A broad range of interventions will potentially be eligible, which may differ in terms of how the service is organised and what is done to or for the older person. Interventions may meet our criteria for including two or more interacting components by including multiple discrete practices, such as exercise sessions and nutritional advice. Other eligible interventions could include one practice that interacts with other structural elements such as being reliant on general practice or other services;

For peer review only - http://bmjopen.bmj.com/site/about/guidelines.xhtml

or interaction with contextual factors by being substantially tailored to the person's physical and social environment. Examples would include comprehensive geriatric assessment or rehabilitation interventions.

Interventions that would not be considered eligible for inclusion are as follows.

- The intervention is either not initiated, or not mainly provided, in the community, or neither. For example, interventions delivered in outpatient, day hospital, inpatient, and intermediate (post-acute) care settings.
- The intervention includes only one discrete component (intervention practices, structural elements and contextual factors) such as a drug, treadmill training, yoga, provision of information, cataract surgery, hearing aid, medication review, nutritional supplements.
- The intervention is not targeted at the individual person, with provision of appropriate specialist care. For example, general staff education (not training in a patient-level intervention), practice-level reorganisation, operational, managerial or IT interventions, public health messages.
- A focus of the intervention is not sustaining (maintaining or improving) independence in activities of daily living. For example, interventions that primarily address cognitive deficits, mood disorders, or both will be excluded, unless they also aimed to improve overall independence.
- Condition-specific interventions, for example case management for older people with diabetes, COPD or depression.
- Interventions in which the primary focus is falls prevention as this evidence is already well synthesised, including in a recent NMA.[18] Nonetheless, falls will be a key additional outcome in this review.

Comparator: usual care, "placebo" or attention control, or a different complex intervention meeting our criteria.

Outcome(s): Studies will be included where outcome data were recorded at a minimum 24-week timepoint. For all outcomes of interest, data will be extracted and categorised for three timepoints: around 6 months, around 12 months, and around 24 months.

Main outcomes

– The main outcomes are: living at home (defined either as a reported trial outcome, or the inverse of care home admission and mortality if reported separately); activities of daily living (basic/instrumental); home-care services (non-healthcare professional) usage; hospitalisation; care home admission; costs; and cost-effectiveness.

For peer review only - http://bmjopen.bmj.com/site/about/guidelines.xhtml

Additional outcomes

– The additional outcomes are: health status/health-related quality of life, depression, loneliness, falls, mortality.

This update to the landmark 2008 systematic review and meta-analysis by Beswick and colleagues[11] refines the criteria used by that review, which will lead to the exclusion of some of their included studies; we recount these differences here. We will exclude falls prevention studies as a recent network meta-analysis has been conducted in that area.[18] Our criteria exclude interventions that are initiated in hospital and those conducted in outpatient settings, to ensure the interventions are firmly placed in the community. We will also exclude interventions in residential care settings, as these are already the subject of large-scale reviews, and the different settings provide different opportunities and challenges for intervening. Finally, we will exclude studies without an intervention targeted at the older person, for example financial incentives for general practitioners, for consistency within our network meta-analysis.

Search strategy

Search strategies have been developed and tested through an iterative process by an experienced medical information specialist in consultation with the review team. We will search the following databases from inception:

- Cochrane Central Register of Controlled Trials (CENTRAL; issue 9 of 12, September 2020);
- MEDLINE Ovid (1946 to September 2020);
- Embase and Embase Classic Ovid (1947 to September 2020);
- CINAHL EBSCO (1981 to September 2020); and
- PsycINFO Ovid (1806 to September 2020).

We will also search trial registers (ClinicalTrials.gov and the International Clinical Trials Registry) from inception and scan reference lists of included papers. Publication status, date or language restrictions will not be used, and translation will be arranged as necessary throughout the process. A draft search strategy for MEDLINE is provided in the *Supplementary Appendix A*. A Preferred Reporting Items for Systematic Reviews and Meta-Analyses (PRISMA) flow chart will be presented showing the process of study selection (Supplementary Figure S1).[14]

Study selection

Following deduplication, search results will be imported into the Rayyan web application (https://rayyan.qcri.org/). Two researchers will independently assess the title and abstract of each record. We will obtain full text articles for all potentially eligible trials. Study selection will be

For peer review only - http://bmjopen.bmj.com/site/about/guidelines.xhtml

conducted by two researchers with guidance from the project management group (PMG), and disagreements will be resolved by consensus discussion involving the PMG. We will contact study authors if further information is required.

Data collection process

Two researchers will independently extract data using a piloted data extraction form in a purpose-built Microsoft Access database. Characteristics of included and excluded studies tables will be produced in Review Manager (RevMan) [Computer program] Version 5.4. Summary of findings tables will be produced in GRADEPro.[19]

For peer review only

Intervention grouping

We will group interventions for NMA in a three-stage process.

1. We will use the Template for Intervention Description and Replication (TIDieR) framework to summarise reported interventions (including comparators).[20] The TIDieR framework includes 12 key items including the why, what, who provided, how, where, when and how much of the intervention including the broader healthcare context.
2. We will complete a content analysis of the summarised interventions using the TIDieR framework in nVivo 12 to inform provisional groupings.[21]
3. We will develop provisional intervention groupings based upon the service organisation or structure (e.g., team structure), key patient care processes (e.g., assessment, follow-up), and specific patient care interventions (e.g., exercise, ADL practice, relaxation). The intervention types will become the nodes in the NMA.

Assessment of frailty

We anticipate that a range of validated instruments and operationalised measures will be used to identify pre-frailty and frailty in included trial populations of some studies. Examples of such frailty measures include: the use of the Fried phenotype model, the Tilburg Frailty Indicator, Groningen Frailty Indicator, Study of Osteoporotic Fractures criteria, Chinese Canadian study of health and aging clinical frailty scale, Hebrew Rehabilitation Center for Aged Vulnerability Index, Vulnerable Elders Survey, Brief frailty measure derived from the Canadian study of health and aging or a formally produced Frailty Index.[22] We will classify the trial population in accordance with the frailty measure, so long as it is developed or validated according to the modern meaning of frailty and not as a generic term for being old or disabled. We will report methods used for each trial, including cut-points for identification of pre-frailty and frailty.

For peer review only - http://bmjopen.bmj.com/site/about/guidelines.xhtml

We also anticipate that many studies will not formally have described study populations in terms of frailty. In such circumstances two reviewers with extensive clinical academic frailty expertise (AC & JG) will independently use the well-validated phenotype model as a framework to categorise study-level frailty profile (not frail; pre-frailty; frailty) of trial participants if the relevant variables are reported.[23] The model is based on five characteristics (weight loss; exhaustion; low energy expenditure; slow gait speed; low grip strength). Evidence of ≥3 indicates frailty, 1-2 pre-frailty and 0 not frail. In the remaining studies where neither a recognised frailty measure nor the variables needed to apply the frailty phenotype categorisation are reported, the two reviewers will independently attempt to classify the populations based on trial eligibility criteria and/or reported baseline characteristics closely linked to frailty including gait speed, hand grip strength, mobility, activity, or disability levels. Any disagreements will be resolved by consensus.

In categorising study level frailty, we recognise that trials may include participants across different frailty categories, so as well as 'not frail', 'pre-frail' and 'frail', our categories will also include 'not frail and pre-frail', 'pre-frail and frail' and 'all'.

Our main analysis of the impact of frailty will only include trials that used a validated measure. Trials in which the reviewers allocated a study-level frailty level on the basis of eligibility criteria and/or baseline characteristics will be examined in secondary analyses.

Intention to treat and missing data

If both per-protocol and intention-to-treat analyses are reported for a trial, we will prioritise intention-to-treat data.[24] In all instances, we will report whether analysis was conducted on data that were complete, complete after imputation or incomplete, and we will examine and report any material differences in results across these types. When results for main outcomes are missing for a trial, we will contact authors to request the missing data.

Risk of bias within individual studies

Researchers will independently assess the risk of bias (RoB) of each result of interest from each included trial, using Cochrane's RoB 2 - a revised tool for assessing risk of bias in randomised trials.[24] For cRCTs, we will additionally assess identification/recruitment bias, and the other issues such as loss of clusters, detailed in version 6 of the Cochrane Handbook.[25] For each domain in the RoB 2 tool, a judgement of high risk of bias, low risk of bias, or some concerns will be made, then an overall risk-of-bias judgement will be reached for each assessed outcome, with any disagreements resolved by consensus.

Summary measures

For peer review only - http://bmjopen.bmj.com/site/about/guidelines.xhtml

For each trial and each outcome separately, effect estimates, and confidence intervals (CI) will be extracted comparing intervention and control groups. For continuous outcomes, we aim to extract the intervention effect as mean differences. We will consider using standardised mean difference if different measures are used for similar constructs. For binary outcomes, we will calculate risk ratios (RR) and odds ratios (OR). For survival (time-to-event) outcomes, hazard (rate) ratios will be extracted. Any details about non-proportional hazards will also be extracted. Outcomes at all timepoints will be recorded and grouped appropriately (around 6 months, around 12 months, and around 24 months). Where effect estimates and/or confidence intervals are not available, we will use other information (e.g., p-values, means for each group at follow-up, etc) to derive the information indirectly.

Unit of analysis issues

We will apply adjustment for trials that use cluster randomisation without adjusting standard errors.[25] As intra-class correlations needed to make such correction are rarely reported, we will use values obtained from external literature for the outcome examined (or if these are not available, use a single plausible value, and examine the impact of varying this value in sensitivity analysis).

Examination of potential effect modifiers

Treatment effect modifiers relate to methodological or clinical characteristics of the trials that influence the magnitude of treatment effects (on a given scale), and may include follow-up length, outcome definitions, trial quality (risk of bias), analysis and reporting standards (including risk of selective reporting), and the participant-level characteristics (e.g., leading to trial differences in case-mix variation including frailty). When such effect modifiers are systematically different in trials making the same comparison(s), this manifests itself as between-study heterogeneity in treatment effects. When such effect modifiers are systematically different in the subsets of trials providing direct and indirect evidence about a particular comparison, this causes inconsistency (i.e., disagreement between the direct and indirect evidence for that comparison) in the NMA.

Hence, before any analysis, the distribution of potential effect modifiers will be examined across the studies to inform inconsistency concerns (whether direct and indirect evidence in the NMA are likely to coherent) and whether some trials should be removed to improve consistency. Clearly, such decisions will also be based on the inclusion and exclusion criteria for the project.

Data synthesis

We will meta-analyse the extracted effect estimates using modules within R and Stata, such as *metafor*, *metan*, *mvmeta* and *network*. Random-effects meta-analyses will be conducted, to allow for potential between-study heterogeneity in each intervention effect.[26] Restricted maximum

For peer review only - http://bmjopen.bmj.com/site/about/guidelines.xhtml

likelihood (REML) estimation will be used to fit all the models, with 95% confidence intervals (CIs) derived using an approach to account for uncertainty in the estimate of heterogeneity (tau-squared), such as the Hartung-Knapp Sidik-Jonkman approach.[27] Initially for each outcome, we will perform a separate meta-analysis for each type of intervention, to provide summary effectiveness results based only on direct evidence. We will summarise odds ratios (ORs) and risk ratios (RRs) for binary outcomes, pooled (standardised) mean differences for continuous outcomes, and pooled hazard ratios (HRs) for survival outcomes. We will display forest plots, with study-specific estimates, confidence intervals and weights, alongside the summary (pooled) meta-analysis estimates, 95% CI, and (if appropriate) a 95% prediction interval.

Network meta-analysis (NMA)

An NMA will then be conducted (for each outcome separately), using a multivariate random-effects meta-analysis framework via the *network* module in Stata and using REML estimation (with confidence intervals derived accounting for uncertainty of variance estimates).[28] Nodes in the network will correspond to each intervention group as outlined previously. The NMA framework allows both direct and indirect evidence to contribute toward each intervention effect (treatment contrast), via a consistency assumption.[29] The within-study correlation of multiple intervention effects from the same trial (i.e., in multigroup trials) will be accounted for, and a common between-study variance assumed for all treatment contrasts in the network (thus implying a +0.5 between-study correlation for each pair of treatment effects). If possible, sensitivity to relaxing this assumption will be examined using model fit statistics. We will produce summary (pooled) effect estimates for each pair of treatments in the network, with 95% CI, and the borrowing of strength statistic to reveal the contributions of indirect evidence. For binary outcomes, if possible, we will do an NMA of both OR and RR, to check the robustness of conclusions to the choice of effect measure. Based on the results, the ranking of intervention types will be calculated using resampling methods, and quantified by the probabilities of being ranked first, second, ..., last, together with the mean rank and the Surface Under the Cumulative RAnking curve (SUCRA), with appropriate plots will be presented.

Assessment of inconsistency

The consistency assumption will be examined for each treatment comparison where there is direct and indirect evidence (seen as a closed loop within the network plot). This involves estimating direct and indirect evidence, and comparing the two.[30-32] The consistency assumption will also be examined across the whole network using 'design-by-treatment interaction' models, which allow an overall significance test for inconsistency. If evidence of inconsistency is found, explanations will be sought and resolved (e.g., with consideration of the distribution of effect modifiers; see earlier).

For peer review only - http://bmjopen.bmj.com/site/about/guidelines.xhtml

Examination of small-study effects

If there are 10 or more studies in a meta-analysis, funnel plots will be presented to examine small-study effects (potential publication bias). Egger's, Peter's and Debray's test of asymmetry will be used for continuous, binary and survival outcomes, respectively.

Examination of frailty impact

Meta-analysis (MA) results will initially be presented for all levels combined, then for frailty/pre-frailty where reported data permit. That is, a separate MA and NMA for each frailty type will be conducted. Indeed, this may also reduce any inconsistency (see above). We will also consider extending the MA and NMA models to a meta-regression, with frailty/pre-frailty as a study-level categorical covariate allowing effects of frailty/pre-frailty to vary for each treatment effect, to quantify if intervention effects vary according to population-level frailty.

All analyses to examine frailty impact will initially be restricted to trials using a validated measure. Sensitivity analyses will 1) be restricted to trials using the phenotype model to identify pre-frailty/frailty as an internationally-established reference standard, 2) include trials that used either a validated or an operationalised measure of frailty, 3) include all trials, including by study-level categorisation of frailty status.

Additional analyses

We will also run additional sensitivity analyses to present results of more recent evaluations, restricted to trials in the last 15 years. Meta-regression will be used to quantify differences in summary effects between studies at low risk of bias and other studies, and between those with shorter and longer lengths of follow-up. A multivariate network meta-analysis will be considered to accommodate all outcomes simultaneously, to examine if conclusions remain the same after accounting for the correlation amongst outcomes.[33] As mentioned, we will consider how relaxing the assumption of common between-study variances improves model fit.

Confidence in cumulative evidence

We will use the Grading of Recommendations Assessment, Development and Evaluation (GRADE) framework, adapted for NMA, to rate evidence quality.[34-37] Our assessment of quality of treatment effects will enable generation of GRADE evidence profiles for our individual intervention groupings for each outcome separately.

For peer review only - http://bmjopen.bmj.com/site/about/guidelines.xhtml

The assessment of quality of treatment effects will include presentation and rating of the quality of direct and indirect treatment estimates separately and combined in NMA[36], with a focus on first order loops for assessment of indirect treatment estimates. As we will include RCTs and cRCTs the starting point will be a high-quality evidence rating. For each estimate of treatment effect, we will assess risk of bias, inconsistency, indirectness, imprecision and publication bias. We will make an overall judgement on whether the quality rating for each effect warrants downgrading on the basis of limitations in each of the domains, aligned with GRADE guidance[37]. We will not consider imprecision when rating the direct and indirect estimates to inform the combined NMA rating, aligned with recent guidance.[34] Furthermore, in the presence of incoherence between direct and indirect estimates, we will assess the certainty of evidence of each estimate to guide whether or not the network estimate is downgraded.[34]

Patient and public involvement

Our established patient and public involvement Frailty Oversight Group (FOG) provides connections to the whole spectrum of older people, with a focus on those living with frailty. We have consulted our FOG throughout the development of this protocol and discussed plans in detail at quarterly meetings. A specific example of their influence is our selection of main and additional outcomes of importance for older people. We plan to involve FOG in our intervention grouping and dissemination of this review.

Timelines

Formal screening of search results began in January 2020. Data extraction began in May 2020. Risk of bias assessment and data extraction have not yet begun. We are currently updating our searches (September 2020). The project is due to complete in October 2021.

Ethics and dissemination

Ethics approval is not needed as this systematic review will use aggregated, anonymised data that is available in the public domain.

This will be the first systematic review with network meta-analysis comparing the effectiveness of community-based complex interventions to sustain independence for older people, including the effect of frailty and pre-frailty. The review will use a detailed analysis to group the included interventions to identify the best configurations. Further, it will also review the quality of evidence using the GRADE approach.[36] We will disseminate the findings widely through communication with healthcare providers, conference presentations and academic publications. We will adhere to PRISMA-NMA reporting guidelines.[14] Hence, this systematic review will produce transparent and accessible results that are of great relevance and applicability for a wide audience including

For peer review only - http://bmjopen.bmj.com/site/about/guidelines.xhtml

policymakers, commissioners, health/social care professionals, older people and researchers working with an older population.

Acknowledgements

We are grateful to our Information Specialist, Deirdre Andre, for her assistance developing the search strategy.

Footnotes

Authors' contributions: AC and TFC were responsible for conception and design of the study. RDR, JE, and RB designed the statistical analysis plan. NL, RB, MJ, EP, RR, AF, JRFG provided critical revisions of all aspects of the review. AC and TFC are guarantor of the review. All authors have reviewed and approved the final manuscript.

Funding: This project is funded by the National Institute for Health Research (NIHR) Health Technology Assessment programme (project reference NIHR128862). AC is part funded by the NIHR Applied Research Collaboration, Yorkshire & Humber, and Health Data Research UK, an initiative funded by UK Research and Innovation Councils, NIHR and the UK devolved administrations, and leading medical research charities. AF receives funding from an NIHR Senior Investigator award. The views expressed are those of the author(s) and not necessarily those of the NIHR or the Department of Health and Social Care.

Disclaimer: The funders had no role in the design of the planned study or preparation of this protocol.

Competing interests: The authors declare that they have no competing interests.

Abbreviations: CGA: comprehensive geriatric assessment; GRADE: Grading of Recommendations, Assessment, Development and Evaluation; MA: Meta-analysis; NIHR: National Institute of Health Research; NHS: National Health Service; NMA: Network meta-analysis; PICOS: Population, intervention, comparator, outcomes, study design; PRISMA-P: Preferred Reporting Items for Systematic review and Meta-Analysis Protocols; RoB: Risk of bias; SUCRA: Surface Under the Cumulative RAnking curve; TIDieR: Template for Intervention Description and Replication

For peer review only

For peer review only - http://bmjopen.bmj.com/site/about/guidelines.xhtml

References

1. United Nations. Department of Economic and Social Affairs, Population Division. World Population Prospects 2019 [Available from: https://population.un.org/wpp/ accessed 24/09/2020.
2. Overview of the UK population: November 2018. Office for National Statistics, London, UK.
3. Government of United Kingdom. The Grand Challenge missions: Ageing society 2019 [updated 13/09/2019. Available from: https://www.gov.uk/government/publications/industrial-strategy-the-grand-challenges/missions accessed 23/09/2020.
4. World Health Organization. Decade of Healthy Ageing: 2020-2030 [Available from: https://www.who.int/ageing/decade-of-healthy-ageing accessed 24/09/2020.
5. Gabriel Z, Bowling ANN. Quality of life from the perspectives of older people. *Ageing and Society* 2004;24(5):675-91. doi: 10.1017/S0144686X03001582 [published Online First: 08/24]
6. Clegg A, Young J, Iliffe S, et al. Frailty in elderly people. *Lancet* 2013;381(9868):752-62. doi: 10.1016/S0140-6736(12)62167-9
7. Collard RM, Boter H, Schoevers RA, et al. Prevalence of frailty in community-dwelling older persons: a systematic review. *J Am Geriatr Soc* 2012;60(8):1487-92. doi: 10.1111/j.1532-5415.2012.04054.x
8. UK health and social care spending, 2016. Office for Budget Responsibility. London, UK.
9. Care for older people. Projected expenditure to 2022 on social care and continuing health care for England's older population. Nuffield Trust 2012. London, UK.
10. Han L, Clegg A, Doran T, et al. The impact of frailty on healthcare resource use: a longitudinal analysis using the Clinical Practice Research Datalink in England. *Age Ageing* 2019;48(5):665-71. doi: 10.1093/ageing/afz088 [published Online First: 2019/07/13]
11. Beswick AD, Rees K, Dieppe P, et al. Complex interventions to improve physical function and maintain independent living in elderly people: a systematic review and meta-analysis. *Lancet* 2008;371(9614):725-35. doi: 10.1016/S0140-6736(08)60342-6
12. Stuck AE, Egger M, Hammer A, et al. Home visits to prevent nursing home admission and functional decline in elderly people: systematic review and meta-regression analysis. *Jama* 2002;287(8):1022-8.
13. Tappenden P, Campbell F, Rawdin A, et al. The clinical effectiveness and cost-effectiveness of home-based, nurse-led health promotion for older people: a systematic review. *Health Technol Assess* 2012;16(20):1-72. doi: 10.3310/hta16200
14. Hutton B, Salanti G, Caldwell DM, et al. The PRISMA Extension Statement for Reporting of Systematic Reviews Incorporating Network Meta-analyses of Health Care Interventions: Checklist and Explanations. *Annals of Internal Medicine* 2015;162(11):777-84. doi: 10.7326/m14-2385 %m 26030634

For peer review only - http://bmjopen.bmj.com/site/about/guidelines.xhtml

15. Moher D, Shamseer L, Clarke M, et al. Preferred reporting items for systematic review and meta-analysis protocols (PRISMA-P) 2015 statement. *Systematic Reviews* 2015;4(1):1. doi: 10.1186/2046-4053-4-1
16. Crocker T, Forster A, Young J, et al. Physical rehabilitation for older people in long-term care. *Cochrane Database Syst Rev* 2013(2):CD004294. doi: 10.1002/14651858.CD004294.pub3
17. Low L-F, Fletcher J, Goodenough B, et al. A Systematic Review of Interventions to Change Staff Care Practices in Order to Improve Resident Outcomes in Nursing Homes. *PLOS ONE* 2015;10(11):e0140711. doi: 10.1371/journal.pone.0140711
18. Tricco AC, Thomas SM, Veroniki AA, et al. Comparisons of Interventions for Preventing Falls in Older Adults: A Systematic Review and Meta-analysis. *JAMA* 2017;318(17):1687-99. doi: 10.1001/jama.2017.15006
19. GRADEpro GDT: GRADEpro Guideline Development Tool [Software]. McMaster University, 2020 (developed by Evidence Prime, Inc.). Available from gradepro.org. [accessed 06/10/2020.
20. Hoffmann TC, Glasziou PP, Boutron I, et al. Better reporting of interventions: template for intervention description and replication (TIDieR) checklist and guide. *BMJ* 2014;348:g1687. doi: 10.1136/bmj.g1687 [published Online First: 2014/03/13]
21. Patton M. Qualitative research and evaluation methods: integrating theory and practice. 4th edition. SAGE publishing, Los Angeles2015.
22. Crocker TF, Brown L, Clegg A, et al. Quality of life is substantially worse for community-dwelling older people living with frailty: systematic review and meta-analysis. *Quality of Life Research* 2019;28(8):2041-56. doi: 10.1007/s11136-019-02149-1
23. Fried LP, Tangen CM, Walston J, et al. Frailty in older adults: evidence for a phenotype. *J Gerontol A Biol Sci Med Sci* 2001;56(3):M146-56. [published Online First: 2001/03/17]
24. Sterne JAC, Savović J, Page MJ, et al. RoB 2: a revised tool for assessing risk of bias in randomised trials. *BMJ* 2019;366:l4898. doi: 10.1136/bmj.l4898
25. Higgins JPT, Thomas J, Chandler J, et al., editors. *Cochrane Handbook for Systematic Reviews of Interventions*. 2nd ed. Chichester, UK: Cochrane Collaboration and John Wiley & Sons, 2019.
26. Riley RD, Higgins JP, Deeks JJ. Interpretation of random effects meta-analyses. *BMJ* 2011;342:d549. doi: 10.1136/bmj.d549
27. Langan D, Higgins JPT, Jackson D, et al. A comparison of heterogeneity variance estimators in simulated random-effects meta-analyses. *Res Synth Methods* 2018 doi: 10.1002/jrsm.1316
28. Riley RD, Jackson D, Salanti G, et al. Multivariate and network meta-analysis of multiple outcomes and multiple treatments: rationale, concepts, and examples. *BMJ* 2017;358:j3932. doi: 10.1136/bmj.j3932
29. Dias S, Ades AE, Welton NJ, et al. Network meta-analysis for decision-making: John Wiley & Sons 2018.

For peer review only - http://bmjopen.bmj.com/site/about/guidelines.xhtml

30. Dias S, Welton NJ, Caldwell DM, et al. Checking consistency in mixed treatment comparison meta-analysis. *Stat Med* 2010;29(7-8):932-44. doi: 10.1002/sim.3767
31. Dias S, Welton NJ, Sutton AJ, et al. Evidence synthesis for decision making 4: inconsistency in networks of evidence based on randomized controlled trials. *Med Decis Making* 2013;33(5):641-56. doi: 10.1177/0272989X12455847
32. Veroniki AA, Vasiliadis HS, Higgins JP, et al. Evaluation of inconsistency in networks of interventions. *Int J Epidemiol* 2013;42(1):332-45. doi: 10.1093/ije/dys222
33. Jackson D, Bujkiewicz S, Law M, et al. A matrix-based method of moments for fitting multivariate network meta-analysis models with multiple outcomes and random inconsistency effects. *Biometrics* 2018;74(2):548-56. doi: 10.1111/biom.12762 [published Online First: 2017/08/15]
34. Brignardello-Petersen R, Bonner A, Alexander PE, et al. Advances in the GRADE approach to rate the certainty in estimates from a network meta-analysis. *J Clin Epidemiol* 2018;93:36-44. doi: 10.1016/j.jclinepi.2017.10.005 [published Online First: 2017/10/21]
35. Guyatt GH, Oxman AD, Schunemann HJ, et al. GRADE guidelines: a new series of articles in the Journal of Clinical Epidemiology. *J Clin Epidemiol* 2011;64(4):380-2. doi: 10.1016/j.jclinepi.2010.09.011 [published Online First: 2010/12/28]
36. Puhan MA, Schunemann HJ, Murad MH, et al. A GRADE Working Group approach for rating the quality of treatment effect estimates from network meta-analysis. *BMJ* 2014;349:g5630. doi: 10.1136/bmj.g5630 [published Online First: 2014/09/26]
37. Schünemann H, Brożek J, Guyatt G, et al. Handbook for grading the quality of evidence and the strength of recommendations using the GRADE approach. Available from https://gdt.gradepro.org/app/handbook/handbook.html2013.

For peer review only

For peer review only - http://bmjopen.bmj.com/site/about/guidelines.xhtml

Appendix A: MEDLINE search strategy

Database: Ovid MEDLINE(R) and Epub Ahead of Print, In-Process & Other Non-Indexed Citations and Daily <1946 to December 31, 2019>

Search Strategy:

----

1 randomized controlled trial.pt. (497917)

2 controlled clinical trial.pt. (93495)

3 randomized.ab. (465667)

4 placebo.ab. (204054)

5 clinical trials as topic.sh. (189709)

6 randomly.ab. (324606)

7 trial.ti. (210688)

8 or/1-7 (1260622)

9 exp animals/ not humans.sh. (4659772)

10 8 not 9 [Cochrane Highly Sensitive Search Strategy for identifying randomized trials in MEDLINE: sensitivity- and precision-maximizing version (2008 revision] (1159627)

11 Clinical Trial, Phase III/ (16136)

12 ("phase 3" or "phase3" or "phase III" or "P3" or "PIII").ti,ab,kw. (64901)

13 11 or 12 [search filter for phase three trials to suppplement Cochrane HSSS, Cooper 2019] (70728)

14 10 or 13 [final RCT filter] (1199211)

15 (frail* or prefrailty).tw. (19992)

16 exp aged/ (3036862)

17 geriatrics/ (29616)

18 (elder* or older or old people* or old person* or old wom#n*1 or old m#n*1 or old male*1 or old female*1 or old adult*1 or old age* or aging or ageing or geriatric* or senior citizen* or seniors or pensioner* or veteran* or sexagenarian* or septuagenarian* or octogenarian* or nonagenarian* or centenarian*).tw,kf. (1233323)

19 (over adj2 ("60" or "61" or "62" or "63" or "64" or "65" or "66" or "67" or "68" or "69" or "70" or "71" or "72" or "73" or "74" or "75" or "76" or "77" or "78" or "79" or "80" or "81" or "82" or "83" or "84" or "85" or "86" or "87" or "88" or "89" or "90" or "91" or "92" or "93" or "94" or "95" or "96" or "97" or "98" or "99" or "100") adj years).tw. (19080)

20 or/15-19 [older or frail people] (3792854)

21 independent living/ (5105)

22 community health services/ (31116)

23 community health nursing/ (19479)

24 Community support services.tw. (163)

25 exp managed care programs/ (39897)

For peer review only

26 (health maintenance organi?ation* or HMO*).tw. (13072)

27 (Social adj3 services).tw. (9360)

28 Voluntary services.tw. (93)

29 *home nursing/ (5264)

30 House Calls/ (3475)

31 house call*.tw. (615)

32 (home adj5 visit*).tw. (11016)

33 ((general practice or primary care or nurse* or group or ambulatory clinic or geriatric clinic) adj3 visit*).tw. (8476)

34 *geriatric assessment/ (12510)

35 (pharmac* adj2 visit).tw. (182)

36 ((home or house) adj2 appointment*).tw. (42)

37 Home Care Services/ (32888)

38 Home care service*.tw. (1725)

39 *health services for the aged/ (13598)

40 home health nursing/ (319)

41 district nursing.tw. (649)

42 health visit*.ti. or health visit*.ab. /freq=2 (2224)

43 community matron*.ti. or community matron*.ab. /freq=2 (83)

44 (home adj3 (intervention* or support* or assessment*)).tw. (7580)

45 preventive health services/ (13212)

46 ((preventive* or preventative*) adj5 medicine).tw. (6792)

47 preventative medicine/ (11603)

48 ((preventive* or preventative*) adj3 (program* or intervent* or support* or care or service* or approach* or case management or measure* or OT or occupational therapy or assess*)).tw. (57977)

49 or/21-48 [specific interventions] (261556)

50 geriatric nursing/ (13511)

51 geriatric nurs*.tw,kf. (1086)

52 or/50-51 [geriatric nursing] (13877)

53 community.ti,ab,kf. (464179)

54 community health services/ or community health nursing/ or community mental health services/ or community pharmacy services/ (71636)

55 "domiciliary care"/ (32888)

56 aftercare/ (8799)

57 primary health care/ (75131)

For peer review only

58 (domiciliary or (social support and home*) or ((homecare or medical) adj2 home) or (home and package*) or (outreach and home) or (alternative setting and home) or home visit* or home manag* or homecare or home care or home therap* or (model* adj1 home*) or home program* or home monitor*).tw. (53213)

59 ((live or living or lived or dwell*) adj5 ("at home" or "own home" or "in home" or alone or independent*)).tw. (15304)

60 (home-based or homebased or homebound).tw. (10615)

61 (Home care or primary care or primary health care or primary healthcare).tw. (146114)

62 or/53-61 [interventions in a community or home setting] (709468)

63 52 and 62 [geriatric nursing and interventions in a community or home setting] (1982)

64 49 or 63 [all interventions] (262247)

65 (coronary heart disease or CHD or chronic obstructive pulmonary disease or COPD or kidney failure or CKD or Heart failure or diabetes or asthma or cancer or schizophrenia or severe mental illness*).ti. (1420118)

66 64 not 65 [all intervenions excluding specific diseases in title] (247185)

67 14 and 20 and 66 [RCTS and older people and interventions] (6170)

For peer review only

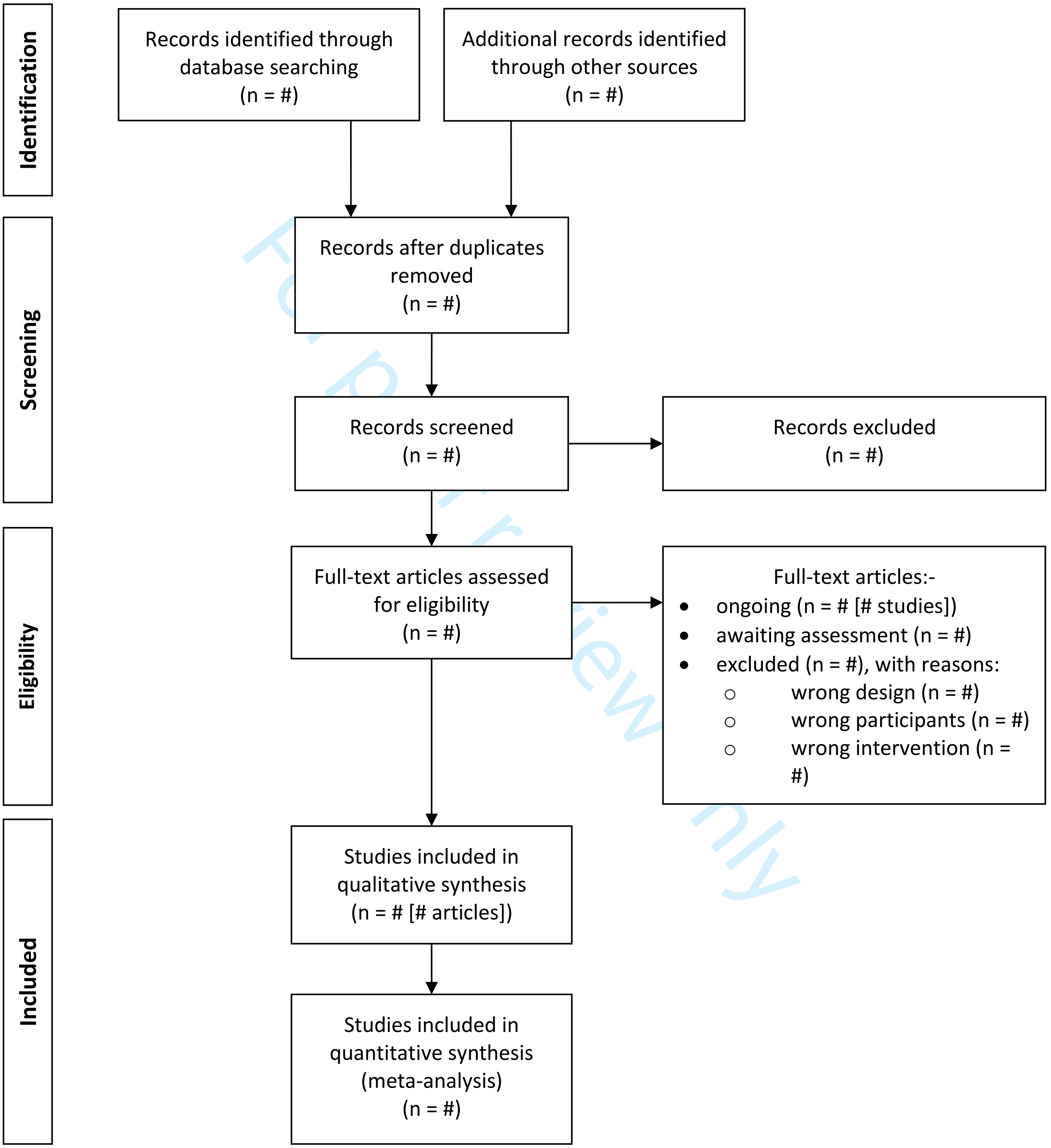

For peer review only - http://bmjopen.bmj.com/site/about/guidelines.xhtml

Additional file 1: PRISMA-P checklist

PRISMA-P (Preferred Reporting Items for Systematic review and Meta-Analysis Protocols) 2015 checklist: recommended items to address in a systematic review protocol*

Section and topic	Item No	Checklist item	(Page No.#)
**ADMINISTRATIVE INFORMATION**			
Title:			
Identification	1a	Identify the report as a protocol of a systematic review	1
Update	1b	If the protocol is for an update of a previous systematic review, identify as such	NA
Registration	2	If registered, provide the name of the registry (such as PROSPERO) and registration number	3
Authors:			
Contact	3a	Provide name, institutional affiliation, e-mail address of all protocol authors; provide physical mailing address of corresponding author	1-2
Contributions	3b	Describe contributions of protocol authors and identify the guarantor of the review	16
Amendments	4	If the protocol represents an amendment of a previously completed or published protocol, identify as such and list changes; otherwise, state plan for documenting important protocol amendments	NA
Support:			
Sources	5a	Indicate sources of financial or other support for the review	16
Sponsor	5b	Provide name for the review funder and/or sponsor	
Role of sponsor or funder	5c	Describe roles of funder(s), sponsor(s), and/or institution(s), if any, in developing the protocol	
**INTRODUCTION**			
Rationale	6	Describe the rationale for the review in the context of what is already known	5-6
Objectives	7	Provide an explicit statement of the question(s) the review will address with reference to participants, interventions, comparators, and outcomes (PICO)	6
**METHODS**			
Eligibility criteria	8	Specify the study characteristics (such as PICO, study design, setting, time frame) and report characteristics (such as years considered, language, publication status) to be used as criteria for eligibility for the review	7-9
Information sources	9	Describe all intended information sources (such as electronic databases, contact with study authors, trial registers or other grey literature sources) with planned dates of coverage	9

For peer review only

Search strategy	10	Present draft of search strategy to be used for at least one electronic database, including planned limits, such that it could be repeated	Appendix-A
Study records:			
Data management	11a	Describe the mechanism(s) that will be used to manage records and data throughout the review	9-10
Selection process	11b	State the process that will be used for selecting studies (such as two independent reviewers) through each phase of the review (that is, screening, eligibility and inclusion in meta-analysis)	9-10
Data collection process	11c	Describe planned method of extracting data from reports (such as piloting forms, done independently, in duplicate), any processes for obtaining and confirming data from investigators	9-10
Data items	12	List and define all variables for which data will be sought (such as PICO items, funding sources), any pre-planned data assumptions and simplifications	9-10
Outcomes and prioritization	13	List and define all outcomes for which data will be sought, including prioritization of main and additional outcomes, with rationale	8-9
Risk of bias in individual studies	14	Describe anticipated methods for assessing risk of bias of individual studies, including whether this will be done at the outcome or study level, or both; state how this information will be used in data synthesis	11-12
Data synthesis	15a	Describe criteria under which study data will be quantitatively synthesised	12-15
	15b	If data are appropriate for quantitative synthesis, describe planned summary measures, methods of handling data and methods of combining data from studies, including any planned exploration of consistency (such as I^2, Kendall's τ)	
	15c	Describe any proposed additional analyses (such as sensitivity or subgroup analyses, meta-regression)	
	15d	If quantitative synthesis is not appropriate, describe the type of summary planned	
Meta-bias(es)	16	Specify any planned assessment of meta-bias(es) (such as publication bias across studies, selective reporting within studies)	14
Confidence in cumulative evidence	17	Describe how the strength of the body of evidence will be assessed (such as GRADE)	14-15

*** It is strongly recommended that this checklist be read in conjunction with the PRISMA-P Explanation and Elaboration (cite when available) for important clarification on the items. Amendments to a review protocol should be tracked and dated. The copyright for PRISMA-P (including checklist) is held by the PRISMA-P Group and is distributed under a Creative Commons Attribution Licence 4.0.**

From: Shamseer L, Moher D, Clarke M, Ghersi D, Liberati A, Petticrew M, Shekelle P, Stewart L, PRISMA-P Group. Preferred reporting items for systematic review and meta-analysis protocols (PRISMA-P) 2015: elaboration and explanation. BMJ. 2015 Jan 2;349(jan02 1):g7647.

For peer review only
